# Deletion of heat shock protein 60 in adult mouse cardiomyocytes perturbs mitochondrial protein homeostasis and causes heart failure

**DOI:** 10.1038/s41418-019-0374-x

**Published:** 2019-06-17

**Authors:** Feifei Fan, Yaoyun Duan, Feili Yang, Christa Trexler, Hong Wang, Lei Huang, Yali Li, Huayuan Tang, Gang Wang, Xi Fang, Jie Liu, Nan Jia, Ju Chen, Kunfu Ouyang

**Affiliations:** 1 School of Chemical Biology and Biotechnology, State Key Laboratory of Chemical Oncogenomics, Peking University Shenzhen Graduate School, 518055 Shenzhen, China; 2 Department of Medicine, School of Medicine, University of California San Diego, La Jolla, CA 92093 USA; 3 Shenzhen Peking University Hospital, 518055 Shenzhen, China; 4 Department of Pathophysiology, School of Medicine, Shenzhen University, 518055 Shenzhen, China; 5 Shenzhen People’s Hospital, 518055 Shenzhen, China

**Keywords:** Chaperones, Protein quality control, Cardiomyopathies

## Abstract

To maintain healthy mitochondrial enzyme content and function, mitochondria possess a complex protein quality control system, which is composed of different endogenous sets of chaperones and proteases. Heat shock protein 60 (HSP60) is one of these mitochondrial molecular chaperones and has been proposed to play a pivotal role in the regulation of protein folding and the prevention of protein aggregation. However, the physiological function of HSP60 in mammalian tissues is not fully understood. Here we generated an inducible cardiac-specific HSP60 knockout mouse model, and demonstrated that HSP60 deletion in adult mouse hearts altered mitochondrial complex activity, mitochondrial membrane potential, and ROS production, and eventually led to dilated cardiomyopathy, heart failure, and lethality. Proteomic analysis was performed in purified control and mutant mitochondria before mutant hearts developed obvious cardiac abnormalities, and revealed a list of mitochondrial-localized proteins that rely on HSP60 (HSP60-dependent) for correctly folding in mitochondria. We also utilized an in vitro system to assess the effects of HSP60 deletion on mitochondrial protein import and protein stability after import, and found that both HSP60-dependent and HSP60-independent mitochondrial proteins could be normally imported in mutant mitochondria. However, the former underwent degradation in mutant mitochondria after import, suggesting that the protein exhibited low stability in mutant mitochondria. Interestingly, the degradation could be almost fully rescued by a non-specific LONP1 and proteasome inhibitor, MG132, in mutant mitochondria. Therefore, our results demonstrated that HSP60 plays an essential role in maintaining normal cardiac morphology and function by regulating mitochondrial protein homeostasis and mitochondrial function.

## Introduction

Cardiac myocytes are enriched in mitochondria in order to generate the large amount of ATP required to maintain normal cardiac contractile function. This energy supply must be tightly regulated to effectively meet the demands of the heart, especially during periods of increased workload or adrenergic stimulation. In addition to being the core of energy metabolism in the cell, mitochondria also regulate many aspects of intermediate metabolism, calcium buffering, and other cellular processes such as apoptosis [1]. Furthermore, mitochondrial dysfunction has been strongly associated with the onset and progression of various cardiac diseases including dilated cardiomyopathy and heart failure [2, 3].

Although mitochondria possess their own genome, most mitochondrial proteins are actually encoded in the nucleus. More than 1200 proteins exist in mitochondria, but only 13 proteins are encoded by mammalian mitochondrial DNA [4, 5]. Nuclear-encoded mitochondrial precursor proteins have to be maintained in a relatively unfolded state in the cytosol for efficient transportation into the mitochondria via the narrow pores formed by tightly gated translocons [6]. Thereafter, these proteins must be correctly folded inside the mitochondria to avoid unwanted protein-protein interactions or aggregation. Mitochondria have a dedicated repertoire of molecular chaperones located in both the intermembrane space (IMS) and matrix to promote efficient mitochondrial protein folding and complex assembly. The mitochondrial chaperonin heat shock protein 60 (HSP60) consists of both HSP60 (the homologous GroEL *Escherichia coli* protein) and heat shock protein 10 (HSP10, the homologous GroES *Escherichia coli* protein) subunits, which form a barrel-shaped complex to primarily facilitate the folding of relatively small, soluble monomeric proteins [7, 8]. Mitochondrial HSP70 (mtHSP70), HSP90, and an HSP90 homolog Tumor Necrosis Factor Receptor-Associated Protein-1 (TRAP-1), have also been shown to promote protein folding in mitochondria [9–12].

The mitochondrial HSP60/HSP10 complex is composed of two heptameric rings of the large subunit (HSP60) stacked back to back [13, 14]. The importance of this chaperonin complex has been highlighted in *Escherichia coli* and yeast, in which loss of either HSP60 or HSP10 results in a lethal phenotype [8, 15–18]. Knockout experiments also demonstrated that HSP60 is essential for survival in Drosophila [19]. In mammals, however, the physiological roles of HSP60 and HSP10 in vivo have not been well studied. Deletion of HSP60 in mice leads to embryonic lethality shortly after implantation suggesting that HSP60 may be required for cell differentiation and survival during early embryonic development [20]. In humans, two disease-related missense mutations in HSP60 have been associated with a dominant form of hereditary spastic paraplegia and a recessively inherited white matter disorder called MitCHAP60 disease, respectively [21, 22]. In cardiac tissues, it has been shown that HSP60 expression was increased following heat stress and in end-stage heart failure [23, 24]. Furthermore, overexpression of HSP60, HSP10 or both in cultured rat neonatal cardiomyocytes protects cardiac cells from apoptotic cell death induced by simulated ischemia [25], ischemia/reoxygenation [26, 27], or doxorubicin [28]. Although these studies strongly suggested that HSP60 could play an important function in cardiomyocytes, it remains unclear whether HSP60 is required for normal cardiac function in vivo. In the current study, we generated an inducible cardiac-specific HSP60 knockout mouse (HSP60^CKO^) model and demonstrated that deletion of HSP60 in adult mouse cardiomyocytes resulted in dilated cardiomyopathy and heart failure due to abnormal mitochondrial protein homeostasis and function.

## Materials and methods

### Mice

We obtained the *Hsp60* (MGI: 96242) embryonic stem cell clone containing conditional alleles with flanked exon 3 and LacZ-Neo cassettes from the International Knockout Mouse Consortium (EUCOMM ID: 40427). To generate male chimeras we microinjected this construct into blastocysts from C57BL/6 mice (Charles River). Male chimeras were then bred with female Black Swiss mice (Charles River) to generate germline-transmitted heterozygous (*Hsp60*^flox-LacZ-Neo/+^) mice (Fig. S1A). The *Hsp60*^flox-LacZ-Neo/+^ mice were then crossed with B6.Cg-Tg(ACTFLPe)9205Dym/J (FLPe) mice (The Jackson Laboratory) that express a flippase recombinase gene under the control of the human ACTB promoter [29], to remove the LacZ-Neo cassette and obtain *Hsp60* floxed heterozygous (*Hsp60*^f/+^) mice (Fig. S1B). To generate inducible cardiac-specific *Hsp60* knockout mice, *Hsp60*^f/f^ mice were bred with αMHC-MerCreMer (*αMHC-MCM*^+^) transgenic mice that express a tamoxifen-inducible Cre recombinase protein fused to two mutant estrogen receptor ligand binding domains [30]. *αMHC-MCM*^+^Hsp60^f/+^ mice were further crossed with *Hsp60*^f/f^ mice to generate *αMHC-MCM*^+^Hsp60^f/f^ mice. To induce cardiac-specific *Hsp60* gene deletion, 7–8-week-old male *αMHC-MCM*^+^Hsp60^f/f^ mice were intraperitoneally injected with tamoxifen (20 mg/kg/d) for four consecutive days and considered cardiac-specific *Hsp60* knockout (HSP60^CKO^) mice. The littermate *αMHC-MCM*^-^*Hsp60*^f/f^ mice were also treated with tamoxifen using the same procedure and used as control mice.

### DNA analysis

Genomic DNA was extracted from mouse tails as previously described [31], and polymerase chain reaction (PCR) was used to genotype the offspring using the following gene-specific primers (from 5′ to 3′): *Hsp60* (Forward, TGGGTCAAGTACTTTTATCCCCTA; Reverse, GGGAAGGCTAAGACCTACT CATT), αMHC-MerCreMer (Forward, GCCATAGGCTACGGTGTAAAAG; Reverse, TTGGTCAATAAGC CCATCATT).

### Quantitative real-time PCR analysis

Quantitative real-time PCR (qRT-PCR) analysis was performed as previously described [32]. Briefly, total RNA was extracted from heart tissues with TRIzol reagent (Invitrogen) and cDNA was synthesized using *TransScript* One-Step gDNA Removal and cDNA Synthesis SuperMix Kit (Transgen Biotech). qRT-PCR was performed using *TransScript* Tip Green qPCR SuperMix (Transgen Biotech) according to the manufacturer’s instructions with each sample run at least in duplicate. Primer sequences used for qRT-PCR analysis can be found in Supplementary Table 1. Relative transcript abundance was normalized to *Gadph* as previously described [33].

### Immunostaining

Hearts were freshly harvested, fixed in 4% paraformaldehyde diluted in phosphate buffered saline (PBS), incubated with an ascending series of sucrose concentrations from 5 to 20%, and embedded in optimal cutting temperature (OCT) compound (Sakura Finetek USA Inc., Torrance, CA, USA). Cryosections (7 μm) were prepared and immunostaining was performed using cleaved caspase 3 antibody (catalog no. 9661; Cell Signaling Technologies) as previously described [34].

### Histological analysis

Hearts were freshly collected, fixed in 4% paraformaldehyde diluted in PBS, dehydrated by ethanol, cleared in xylene, and embedded in paraffin wax. Serial sections (5 μm) were obtained and stained with hematoxylin and eosin (H&E) as previously described [35]. Masson’s Trichrome staining was performed according to the manufacturer’s protocol (Sigma).

### Mitochondria preparation

Hearts were freshly harvested, washed, minced, and homogenized in 10 volumes (v/w) of Chapel-Perry buffer (in mM: 50 MOPS, 100 KCl, 5 MgSO_4_, 1 EGTA, and 1 ATP, pH 7.4) with a Polytron homogenizer at medium speed (5 s per time, four times). After centrifugation at 200 × *g* for 3 min, the supernatant was collected and centrifuged again at 300 × *g* for 4 min. After that, the supernatant was collected and centrifuged at 8000 × *g* for 17 min. The pellet was then resuspended and represented the mitochondrial fraction while the supernatant was collected as the cytoplasmic fraction.

### Mitochondrial complexes activity assay

The activities of the individual mitochondrial electron transport chain (ETC) complexes I, II, and IV were determined immediately after isolating the mitochondrial fraction from control and HSP10^CKO^ hearts by using ETC complex activity microplate assay kits (complex I, catalog no. ab109721; complex II, catalog no. ab109908; complex III, catalog no. ab109905; complex IV, catalog no. ab109911; Abcam). The signal was quantified using a spectrophotometer according to the protocol provided by the manufacturer. Complex I activity was determined by measuring the oxidation of nicotinamide adenine dinucleotide (NADH) to NAD^+^ represented by the simultaneous reduction of a dye leading to increased absorbance at 450 nm. Complex II activity was measured as the reduction of ubiquinone to ubiquinol by a decrease in absorbance at 600 nm. Complex III activity was measured in a coupled reaction with a mixture solution containing succinate (electron donor of Complex II), oxidized cytochrome c (electron acceptor of complex III), KCN (Complex IV inhibitor) and rotenone (Complex I inhibitor). The rate of coupled Complex II + III reaction was measured by monitoring the conversion of oxidized cytochrome c into the reduced form, which can be observed as an absorbance increase at OD 550 nm. Complex IV activity was determined by following the oxidation of reduced cytochrome c as an absorbance decrease at 550 nm.

### Measurement of mitochondrial membrane potential and ROS production

Isolation of single cardiomyocyte from adult mouse heart by Langendorff perfusion was performed as previously described [36]. Briefly, hearts were perfused with collagenase type II (catalog no. LS004177, Worthington) to dissociate individual cardiomyocytes. Isolated cells were then filtered through a 100-μm mesh nylon filter before performing sequential sedimentation to enrich for myocytes, and plated onto glass-covered dishes coated with Laminin. To measure mitochondrial membrane potential of individual cardiomyocytes, cells were incubated with 25 nM Tetramethylrhodamine (TMRM) (catalog no. T668, Thermofisher Scientific) for 60 min at 37 °C, washed, and imaged by confocal microscopy using the light of 543 nm. To determine mitochondrial superoxide production, cells were incubated with 2 μM MitoSOX™ red mitochondrial superoxide indicator (catalog no. M36008, Thermofisher Scientific) for 20 min at 37 °C, washed, and imaged at 514 nm. Data were analyzed using Image J software.

### Western blotting

Freshly isolated ventricles were homogenized in lysis buffer (20mM Tris-HCl, 20mM NaCl, 0.1mM EDTA, 1% Triton X-100, 0.5% sodium deoxycholate) containing a protease and phosphatase inhibitor cocktail (catalog no. B15001; Biotool, USA). On the other hand, mitochondria were prepared separately as mentioned above and were homogenized in the same lysis buffer to have subcellular mitochondrial fractions. Protein content was measured using the BCA Protein Assay Kit (catalog no. 23227; Pierce, USA) and adjusted for equal loading. Standard procedures were used to run protein samples on SDS-PAGE gels and subsequently transferred to PVDF membranes (Millipore, USA). The primary antibodies utilized in this study can be found in Supplementary Table 2.

### Echocardiography

Echocardiography was performed as previously described [37]. Briefly, mice were anesthetized with 1% isoflurane and underwent echocardiography using the Vevo 2100 ultrasound system (VisualSonics, FUJIFILM, Canada) with a linear transducer 32–55 MHz. Percent fractional shortening (FS) and ejection fraction (EF) were used as indicators of systolic cardiac function. Measurements of heart rate (HR), left ventricular (LV) internal diameter at end-systole (LVIDs) and at end-diastole (LVIDd), LV posterior wall thickness at end-systole (LVPWs) and at end-diastole (LVPWd), and interventricular septal wall thickness at end-systole (IVSs) and at end-diastole (IVSd) were determined from the LV M-mode tracing.

### Quantitative mitochondrial proteomics by LC-MS/MS

Hearts were collected from control and HSP60^CKO^ mice 6 weeks post tamoxifen injection. To minimize the variation of protein expression between animals, mitochondrial fractions isolated from three independent biological replicates were pooled for both control and HSP60^CKO^ samples, and submitted for proteomic analysis (PTM Biolabs, Hangzhou, China). One proteomic analysis per pooled sample was performed. In brief, mitochondrial fractions were sonicated three times on ice using a high intensity ultrasonic processor (Scientz, China) in lysis buffer (8.0M Urea, 10mM DTT, 2mM EDTA and 1% protease inhibitor cocktail III). Protein concentration was determined using the 2-D Quant kit (GE Healthcare Bioscience, USA) according to the manufacturer’s instruction. For digestion, the protein solution was reduced with 10 mM DTT for 1 h at 37 °C, and alkylated with 20;mM iodoacetamide (IAA) in the dark for 45 min at room temperature. Following alkylation, protein samples were diluted to 1 M urea by adding 100mM triethylammonium bicarbonate (TEAB). Finally, trypsin was added at 1:50 trypsin-to protein mass ratio for the first digestion overnight and 1:100 trypsin-to-protein mass ratio for a second 4 h digestion. Approximately 300 μg protein for each sample was digested with trypsin for the following experiments. After trypsin digestion, the peptides were desalted using Strata X C18 SPE columns (Phenomenex, USA) and vacuum-dried. Peptides were reconstituted in 0.5 M TEAB and processed with the 6-plex TMT kit (Thermofisher Scientific, USA) according to the manufacturer’s instruction. After TMT labeling, the sample was fractionated by high pH reverse-phase HPLC using Agilent 300Extent C18 column (5 μm particles, 4.6 mm ID, 250 mm length; Agilent, USA). Peptides were dissolved in 0.1% formic acid (FA), directly loaded onto a reversed-phase pre-column (Acclaim PepMap 100; Thermofisher Scientifc, USA). Peptide separation was performed using a reversed-phase analytical column (Acclaim PepMap RSLC; Thermofisher Scientifc, USA). The gradient was comprised of an increase from 6 to 22% solvent B (0.1% FA in 98% acetonitrile) over 26 min, 22–35% in 8 min, and climbing to 80% in 3min then holding at 80% for the last 3 min, all at a constant flow rate of 300nl/min on an EASY-nLC 1000 UPLC system. The resulting peptides were analyzed by Q Exactive^TM^ Plus hybrid quadrupole-Orbitrap mass spectrometer (Thermofisher Scientific, USA). The resulting MS/MS data were processed using Mascot search engine (V2.3.0). Tandem mass spectra were searched against the *Swissprot Mouse* database. Trypsin/P was specified as the cleavage enzyme allowing up to 2 missing cleavages. Mass error was set to 10 parts per million (ppm) for precursor ions and 0.02 Dalton (Da) for fragment ions. Carbamidomethyl on Cysteine, TMT-6plex (N-term) and TMT-6plex (K) were specified as fixed modifications and oxidation on Methione was specified as variable modifications. FDR was adjusted to <1% and peptide ion score was set ≥20.

### In vitro transcription and translation

Human influenza hemagglutinin (HA) tag (TACCCATACGATGTTCCAGATTACGCT) was added at the C-terminus of the cDNA encoding mouse SIRT3. The constructed fragment was amplified by PCR and subsequently cloned into the vector pSP64 (Promega) at the restriction sites *SmaI* and *HindIII*. Plasmid DNA was added to an aliquot of the TnT^®^ Quick Master Mix (catalog no. L2080, Promega) and incubated in a 50 µl reaction volume for 60–90 min at 30 °C to obtain HA-tagged proteins.

### Mitochondrial protein import and degradation assay

Freshly isolated mitochondria were suspended in import buffer (250 mM sucrose, 5 mM magnesium acetate, 80 mM potassium acetate, 10 mM sodium succinate, 1 mM dithiothreitol, 0.1 mM ADP, 20 mM HEPES-KOH, pH 7.4). Mitochondrial protein concentrations were then measured using the BCA Protein Assay Kit. Subsequently, 50 μg mitochondria were diluted in 200 μl import buffer containing 10 mM ATP, followed by addition of 4 μl HA-tagged preproteins, and incubated at 37 °C for 25 min. Mitochondrial protein import was stopped by addition of valinomycin (2.0 μM) to dissipate mitochondrial membrane potential and subsequent centrifugation at 12,000 × *g* for 5 min at 4 °C. Mitochondrial pellets were then resuspended in 200 μl import buffer containing MG132 (50 μM in DMSO) or vehicle (DMSO), and incubated for 50 min. After that, mitochondria were collected by centrifugation and resuspended in lysis buffer (20mM Tris-HCl, 20mM NaCl, 0.1mM EDTA, 1% Triton X-100, 0.5% sodium deoxycholate) containing protease and phosphatase inhibitor cocktail (catalog no. B15001, Biotool).

### Statistics

Scatter diagrams were drawn using Graphpad Prism 5. Statistical analysis was performed using a two-tailed, unpaired Student’s *t*-test or two-way ANOVA with Bonferroni post-hoc test for multiple comparisons (Graphpad Prism 5). All data represent mean ± SEM (error bars). *P* < 0.05 was considered statistically significant.

## Results

### Generation of an inducible cardiac-specific Hsp60 knockout mouse model

**Fig. 1:**
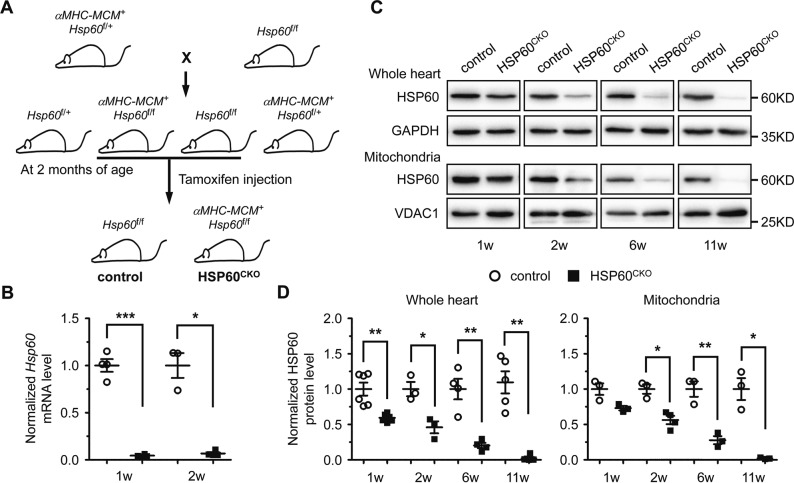
Generation and characterization of inducible cardiac-specific HSP60 knockout mouse model. **a** Schematic diagram of mouse breeding strategy to generate inducible cardiac-specific HSP60 knockout mice. *αMHC-MCM*^+^*Hsp60*^f/f^ mice treated with tamoxifen were used as cardiac-specific HSP60 knockout (HSP60^CKO^) mice. **b** The mRNA levels of *Hsp60* gene in control and HSP60^CKO^ hearts measured by quantitative RT-PCR. *n* = 3–4 mice per group. **c**, **d** Western blot analysis of HSP60 protein in the whole heart tissue and in the mitochondrial fraction (Mito) of control and HSP60^CKO^ hearts at 1 week (1w), 2 weeks (2w), 6 weeks (6w), and 11 weeks (6w) post tamoxifen injection. GAPDH and VDAC1 were used as the loading control for whole heart and mitochondrial proteins, respectively. *n* = 3–5 mice per group. All Data represent mean ± SEM. Significance was determined by two-tailed, unpaired Student’s *t*-test. *p < 0.05, **p < 0.01 versus control. ***p < 0.001 versus control

To understand the physiological role of HSP60 in adult hearts, we generated an inducible cardiac-specific HSP60 knockout mouse model (Fig. S1; Fig. 1a). Seven to 8-week-old male *αMHC-MCM*^+^*Hsp60*^f/f^ mice were treated with tamoxifen to delete *Hsp60* and were used as cardiac-specific *Hsp60* knockout (HSP60^CKO^) mice. In the HSP60^CKO^ hearts, *Hsp60* mRNA levels were dramatically reduced as early as 1 week post tamoxifen injection (Fig. 1b), suggesting that tamoxifen-induced gene deletion was fast and efficient. Furthermore, HSP60 protein levels were almost completely abolished in mutant mice 11 weeks post tamoxifen injection (Fig. 1c, d). However, the decrease in HSP60 protein levels was slower than that of *Hsp60* mRNA expression in HSP60^CKO^ hearts especially at the earlier time points post tamoxifen injection (Fig. 1d), implicating that HSP60 protein in adult cardiomyocytes has a relatively slow turnover rate (Fig. 1b–d). The expression of voltage-dependent anion channel 1 (VDAC1), an outer mitochondrial membrane protein, used as the mitochondrial protein loading control, was not significantly changed between control and mutant hearts (Fig. S2A, B).

### Deletion of HSP60 in adult cardiomyocytes resulted in lethality and cardiac chamber dilation

**Fig. 2:**
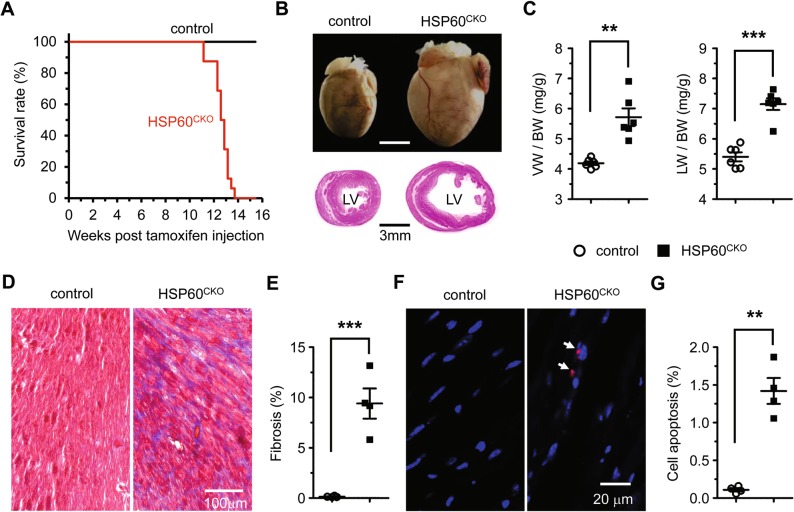
Deletion of HSP60 in adult cardiomyocytes results in mouse lethality and dilated cardiomyopathy. **a** Survival curves of control (*n* = 12) and HSP60^CKO^ (*n* = 16) mice after tamoxifen injection. Significance was determined by the Kaplan–Meier survival analysis. *p* < 0.001 versus control. **b** Representative hearts (top) and H&E stained sections (bottom) of control and HSP60^CKO^ mice 11 weeks post tamoxifen injection. **c** Ratios of ventricle weight to body weight (VW/BW) and lung weight to body weight (LW/BW) in control and HSP60^CKO^ mice 11 weeks post tamoxifen injection. *n* = 6 mice per group. **d** Masson’s trichrome staining of ventricular sections revealed increased fibrosis (blue) in HSP60^CKO^ hearts 11 weeks post tamoxifen injection. **e** Ratios of fibrosis area to the total cell area. *n* = 4 per group. **f** Immunostaining of cell apoptosis marker cleaved caspase 3 (red) in control and HSP60^CKO^ ventricular sections 11 weeks post tamoxifen injection. The sections were counterstained with DAPI (blue) to visualize the nucleus. **g** Ratios of cleaved caspase 3-positive nuclei to the total nuclei. *n* = 4 per group. All Data represent mean ± SEM. Significance was determined by two-tailed, unpaired Student’s *t*-test. **p < 0.01, ***p < 0.001 versus control

In contrast to control animals, all HSP60^CKO^ mice died within 14 weeks post tamoxifen injection (Fig. 2a), suggesting that HSP60 in adult cardiomyocytes is required for the survival of adult mice. The hearts collected from HSP60^CKO^ mice 11 weeks post tamoxifen treatment when the mice started to die were apparently enlarged (Fig. 2b). Histological analysis also revealed dramatic ventricular chamber dilation and ventricular wall thinning in HSP60^CKO^ hearts (Fig. 2b). Consistently, the ratio of ventricular weight to body weight and the ratio of lung weight to body weight were both significantly increased in HSP60^CKO^ mice at this time point (Fig. 2c). This is particularly important since an increase in the latter has regularly been used as a clinical indication of pulmonary edema due to heart failure [38]. Furthermore, mutant hearts displayed severe cardiac fibrosis, as indicated by interstitial collagen deposition (Fig. 2d, e). An increased number of apoptotic cells was also observed in mutant hearts at this time point (Fig. 2f, g), which was associated with increased expression of the proapoptotic protein BAX and reduced expression of the antiapoptotic protein BCL2 (Fig. S3A, B), indicating the activation of mitochondrial apoptotic pathways in mutant hearts.

### HSP60^CKO^ mice developed dilated cardiomyopathy and heart failure

**Fig. 3:**
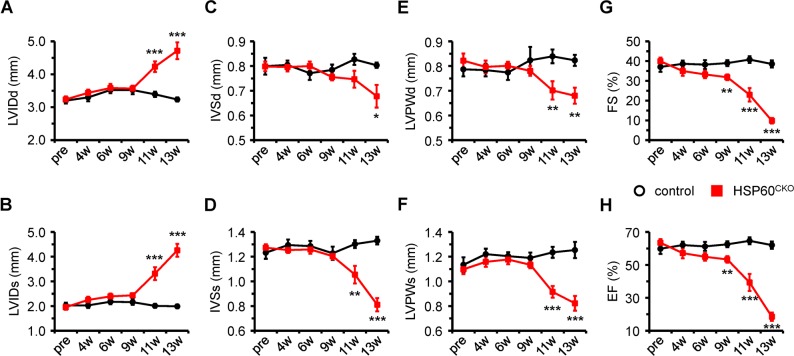
Echocardiographic assessment of cardiac morphology and function in control and HSP60^CKO^ mice. The analysis were performed on left ventricular M-mode in control and HSP60^CKO^ mice prior to (pre), 4 weeks (4w), 6 weeks (6w), 9 weeks (9w), 11 weeks (11w), and 13 weeks (13w) post tamoxifen injection, respectively. **a**, **b** Measurement of left ventricular internal diameter at end-diastole (LVIDd) and at end-systole (LVIDs). **c**, **d** Measurement of interventricular septal wall thickness at end-diastole (IVSd) and at end-systole (IVSs). **e**, **f** Measurement of left ventricular posterior wall thickness at end-diastole (LVPWd) and at end-systole (LVPWs). **g**, **h** Quantitative analysis of left ventricular fractional shortening (FS) and ejection fraction (EF) of control and HSP60^CKO^ hearts. All Data represent mean ± SEM; *n* = 8–12 mice per group. Significance was determined using the two-way ANOVA analysis with Bonferroni post-hoc test. *p < 0.05, **p < 0.01, ***p < 0.001 versus control

We also performed echocardiographic analysis to monitor the changes of cardiac morphology and function. No cardiac morphological and functional changes were observed in HSP60^CKO^ mice prior to 9 weeks after tamoxifen injection (Fig. 3a–h; Fig. S4A). At 9 weeks post tamoxifen injection, the left ventricle (LV) systolic function represented by fractional shortening (FS) and ejection fraction (EF) was slightly decreased in HSP60^CKO^ mice. However, cardiac morphology evidenced by ventricular wall thickness and internal dimension at this time point was not significantly altered in HSP60^CKO^ mice (Fig. 3a–h; Fig. S4B). The mass of mutant hearts and lungs were also not changed at 6 or 9 weeks post tamoxifen injection (Fig. S4C, D). Afterwards, enlarged LV chamber and ventricular wall thinning were found in HSP60^CKO^ mice 11 and 13 weeks after tamoxifen injection, which was consistent with the histological analysis (Fig. 2b). In addition, LV systolic function was further deteriorated in HSP60^CKO^ mice at these two stages, as indicated by further reduces in FS and EF (Fig. 3g, h). All these data demonstrated that HSP60^CKO^ mice developed dilated cardiomyopathy and heart failure, which eventually resulted in the death of mutant mice. It is important to note that treatment of male *αMHC-MCM*^+^*Hsp60*^f/+^ mice with tamoxifen using the same protocol did not cause any lethality (*n* = 12) and cardiac dilation (Fig. S5A-C).

We also assessed the dilated cardiomyopathy in HSP60^CKO^ mice by measuring mRNA levels of cardiac fetal genes including atrial natriuretic factor (*ANF*), brain natriuretic peptide (*BNP*), α-myosin heavy chain (*αMHC*), and β-myosin heavy chain (*βMHC*). A slight increase of *ANF* and *βMHC* expression was observed in HSP60^CKO^ hearts 6 weeks post tamoxifen injection (Fig. S6A-D), implicating that the change of cardiac fetal gene expression precedes alterations in cardiac morphology and function in HSP60^CKO^ hearts. Thereafter, the expression of all four cardiac fetal genes was significantly altered in HSP60^CKO^ hearts 9 weeks and 11 weeks post tamoxifen injection. Furthermore, expression of *collagen1α1*, *collagen3α1*, *Tgfb*, and *cTgf* were also significantly increased in HSP60^CKO^ hearts at these two stages (Fig. S6E-H).

### Loss of HSP60 in cardiomyocytes altered mitochondrial function

**Fig. 4:**
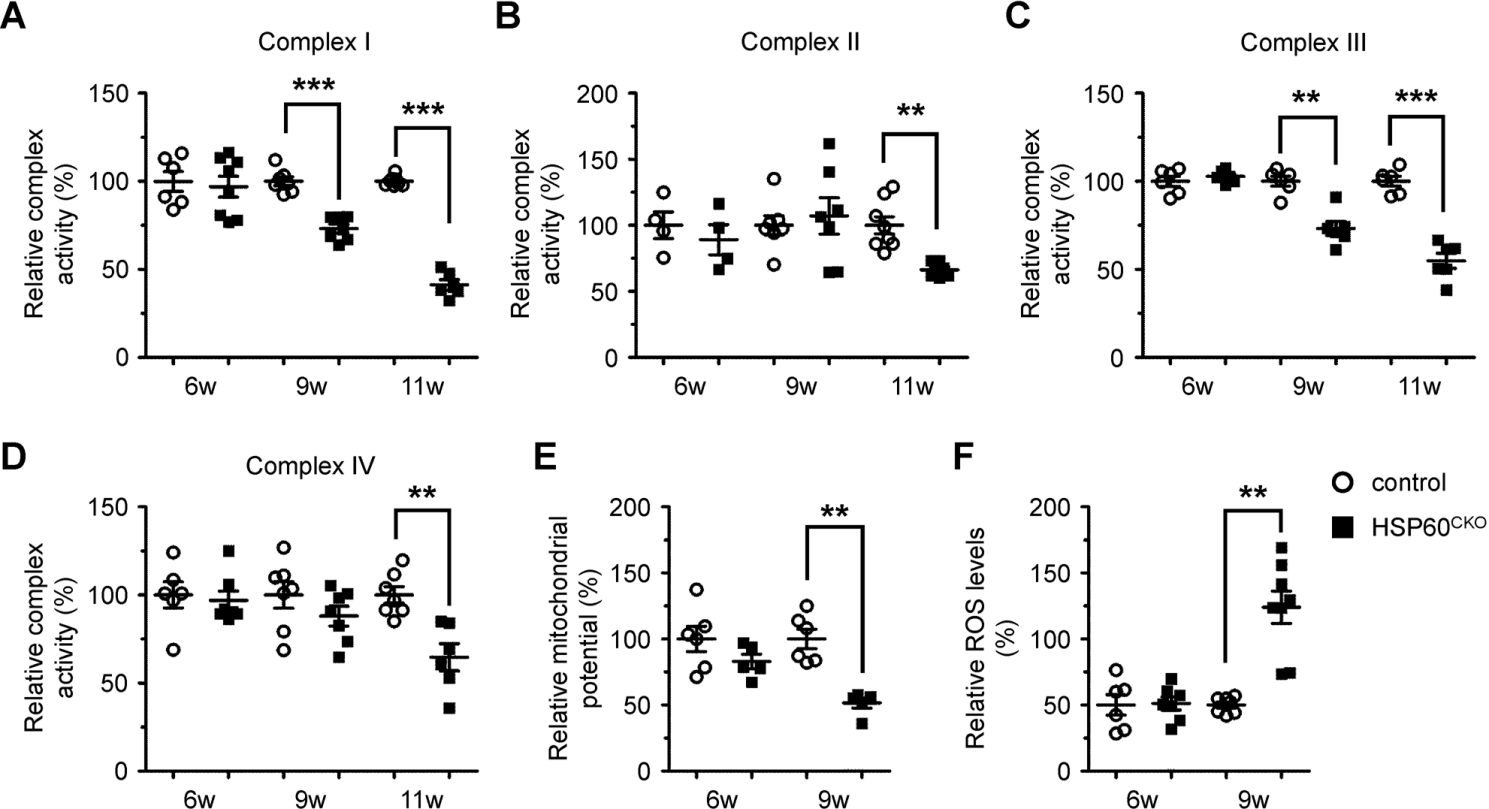
Measurement of mitochondrial functions in control and HSP60^CKO^ hearts. **a–d** Measurement of mitochondrial complex I (**a**), II (**b**), III (**c**), and IV (**d**) enzymatic activities in freshly isolated mitochondria from control and HSP60^CKO^ mice at 6 weeks (6w), 9 weeks (9w) and 11 weeks (11w) post tamoxifen injection. n = 4–7 mice per group. **e** Measurement of mitochondrial membrane potential in cardiomyocytes isolated from control and HSP60^CKO^ hearts 6 weeks and 9 weeks post tamoxifen injection. *n* = 5–6 mice per group. **f** Measurement of mitochondrial ROS levels in cardiomyocytes isolated from control and HSP60^CKO^ hearts 6 weeks and 9 weeks post tamoxifen injection. *n* = 6–7 mice per group. All Data represent mean ± SEM. Significance was determined using the two-way ANOVA analysis with Bonferroni post-hoc test. *p < 0.05, **p < 0.01, ***p < 0.001 versus control

We next examined the effects of HSP60 deletion on mitochondrial functions. The enzymatic activities of all four complexes were measured in freshly isolated mitochondria by spectrophotometric analysis. At 6 weeks post tamoxifen injection, no significant difference was observed in the activities of all four complexes between control and mutant mitochondria (Fig. 4a–d). At 9 weeks post tamoxifen injection, the activities of complex I and complex III in HSP60^CKO^ mitochondria were significantly impaired, whereas the activities of complex II and complex IV were not significantly altered at this time point (Fig. 4a–d). Later on, the activities of all four complexes in HSP60^CKO^ mitochondria were significantly impaired 11 weeks post tamoxifen injection (Fig. 4a–d). We also measured mitochondrial potential and production of mitochondrial reactive oxygen species (ROS). Consistent with the changes in complex activities, reduced mitochondrial potential and increased mitochondrial ROS levels were observed in HSP60^CKO^ cardiomyocytes at 9 weeks post tamoxifen injection but not at 6 weeks post tamoxifen injection (Fig. 4e, f).

### Deletion of HSP60 perturbed mitochondrial protein homeostasis

**Fig. 5:**
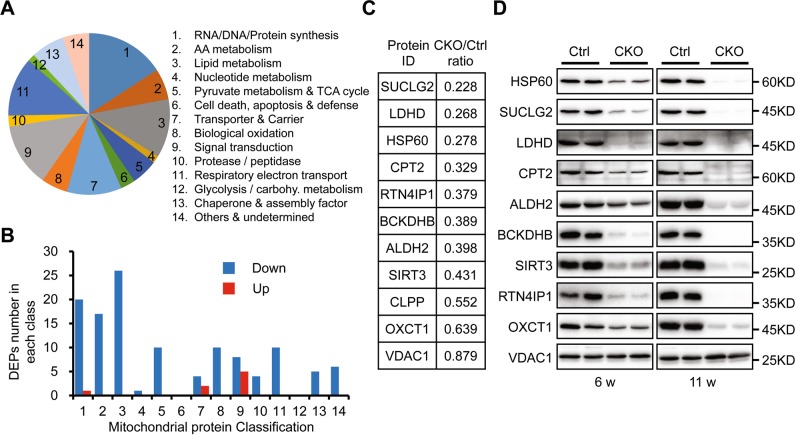
Proteomic analysis and validation of mitochondrial proteins in control and HSP60^CKO^ hearts 6 weeks post tamoxifen injection. **a** Pie chart showing functional classification of mitochondrial-localized proteins characterized from proteomic analysis and distribution of each functional category. **b** Numbers of differentially expression proteins (DEPs) including downregulated (Down) and upregulated (Up) proteins in each functional category. **c** Expression changes of individual mitochondrial proteins in HSP60^CKO^ hearts compared with control hearts revealed by the proteomic analysis. **d** Western blot analysis was used to validate expression changes of individual mitochondrial proteins in HSP60^CKO^ hearts. Mitochondria were isolated from control and HSP60^CKO^ hearts at 6 weeks (6w) and 11 weeks (11w) post tamoxifen injection, and total 10 proteins including HSP60, SUCLG2, LDHD, CPT2, ALDH2, BCKDHB, SIRT3, RTN4IP1, OXCT1, and VDAC1 were analyzed by western blot

To further understand the effects of HSP60 deletion on cardiac mitochondrial protein homeostasis, we performed quantitative proteomic analysis in purified mitochondria prior to the emergence of cardiac morphological and functional defects in mutant mice. In total, 2194 proteins were quantified, among which 676 proteins, accounting for about 30% of total identified proteins, were considered as the mitochondrial-localized proteins according to the online mitochondrial protein database including MitoCarta [5], MitoMiner [39], and the UniProt annotation, while the other detected proteins represented co-purifying contaminants. These 676 mitochondrial proteins could be divided into 14 subgroups according to their functional classification (Fig. 5a), among which 80 proteins were identified as components of mitochondrial electron transport chain (ETC; Table S3). When setting the quantification ratio of >1.5 as the upregulated threshold and <0.67 as the downregulated threshold, 121 mitochondrial-localized proteins were downregulated and only eight mitochondrial-localized proteins were upregulated in HSP60^CKO^ sample relative to the control sample (Table S4), in which most downregulated proteins were involved in processes such as lipid metabolism, amino acid metabolism, and RNA/DNA/protein synthesis (Fig. 5b). Furthermore, 10 ETC proteins were downregulated in mutant sample, all of which belong to ETC complex I (Table S3), implicating that complex I components might be more susceptible to HSP60 deficiency than the other complexes.

Individually, the ratio of HSP60 protein in mutant mitochondria to the control is 0.278 (Fig. 5c), which is very close to 32.4% as revealed by western blotting (Fig. 1d). VDAC1 that was used as the mitochondrial protein loading control was also not different between mutant and control samples (Fig. 5c). Western blotting analysis was further performed to validate the reliability of the proteomic data, and revealed that protein levels of SUCLG2, LDHD, CPT2, ALDH2, BCKDHB, SIRT3, RTN4IP1, and OXCT1 were all significantly decreased in mutant mitochondria at 6 weeks post tamoxifen injection, which was also consistent with the proteomic analysis (Fig. 5c, d; Fig. S7A). Furthermore, these eight proteins were further reduced in mutant mitochondria 11 weeks post tamoxifen injection, which coincided well with the change of HSP60 protein level between these two stages (Fig. 1c). Therefore, these data suggested our proteomic data are highly reliable, further validating our findings about the deleterious effects of HSP60 deletion on mitochondrial protein levels.

### Loss of HSP60 altered mitochondrial protein quality control

**Fig. 6:**
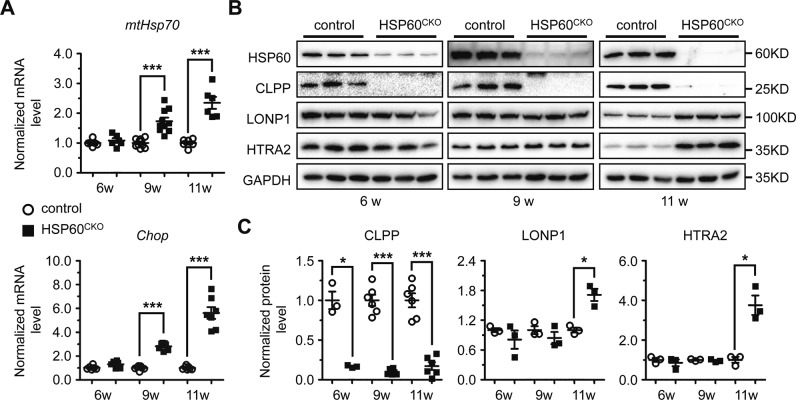
Assessment of mitochondrial unfolded protein response (UPR^mt^) and mitochondrial protease expression in control and HSP60^CKO^ hearts. **a** Mitochondrial UPR^mt^ was assessed by quantitative RT-PCR analysis of *mtHsp70* and *Chop* mRNA levels in control and HSP60^CKO^ hearts at 6 weeks (6w), 9 weeks (9w) and 11 weeks (11w) post tamoxifen injection. *n* = 6–7 mice per group. **b**, **c** Western blotting (**b**) and quantitative analysis (**c**) of mitochondrial proteases including CLPP, LONP1, and HTRA2 in control and HSP60^CKO^ hearts at 6 weeks, 9 weeks and 11 weeks post tamoxifen injection. *n* = 3–6 mice per group. All Data represent mean ± SEM. Significance was determined using the two-way ANOVA analysis with Bonferroni post-hoc test. *p < 0.05, ***p < 0.001 versus control

We further investigated the mechanisms underlying the downregulation of mitochondrial proteins by HSP60 deletion. First, mRNA levels of *Suclg2*, *Ldhd*, *Cpt2*, *Aldh2*, *Bckdhb*, *Sirt3*, *Rtn4ip1*, and *Oxct1* genes were not significantly altered between control and HSP60^CKO^ hearts at 6 weeks post tamoxifen injection (Fig. S7B), suggesting that HSP60 deletion in cardiomyocytes did not change the transcription profile of these genes. We next examined whether HSP60 deletion could result in increases of unfolded or misfolded proteins and subsequent protein degradation. In mitochondria, an increase in unfolded or misfolded proteins will trigger the mitochondrial unfolded protein responses (UPR^mt^) to increase chaperone capacity and re-establish homeostasis within the mitochondrial protein-folding environment [40]. Therefore, we measured the expression of *mtHsp70* and *Chop*, two molecular markers of UPR^mt^ in control and HSP60^CKO^ hearts. However, no significant difference in expression of mt*Hsp70* and *Chop* was found between control and HSP60^CKO^ hearts 6 weeks post tamoxifen injection (Fig. 6a), suggesting that no obvious UPR^mt^ was induced in the mutant hearts at this time point. We also investigated the effects of HSP60 deletion on the expression of mitochondrial proteases. At least two types of ATP-dependent proteases, Lon and ClpXP, are localized in the mitochondrial matrix in various organisms [41, 42]. The level of LONP1, a major member of Lon protease family, was not significantly altered in HSP60^CKO^ hearts 6 weeks post tamoxifen injection (Fig. 6b, c). In addition, the level of HTRA2, a serine protease located in the mitochondrial intermembrane space and released from mitochondria during apoptosis [43, 44], was also not significantly changed in mutant hearts at this time point (Fig. 6b, c). In contrast, the level of CLPP, a proteolytic subunit of the ClpXP protease, was significantly reduced in HSP60^CKO^ hearts at this time point, which is consistent with what we found by proteomic analysis (Fig. 5c; Fig. 6b, c).

We also set up different in vitro systems to assess the effects of HSP60 deletion on mitochondrial protein degradation. Neonatal cardiomyocytes (NCMs) and cardiac fibroblasts (NCFs) were isolated from HSP60^f/f^ mice, cultured and treated with adenovirus-Cre (Adv-Cre). Adv-Cre treatment was able to reduce Hsp60 mRNA levels but not protein levels in NCMs (Fig. S8), probably because HSP60 protein has a relatively long turnover time in NCMs, as is the case in adult cardiomyocytes (Fig. 1c, d). In NCFs, Adv-Cre treatment efficiently reduced both HSP60 mRNA and protein levels (Fig. S9A, B). Furthermore, HSP60 deletion also reduced expression of SIRT3 and SUCLG2 in NCFs (Fig. S9B). More importantly, addition of MG132, an inhibitor of the 26S proteasome also known to enter mitochondria where it can inhibit LONP1 [45, 46], significantly rescued the downregulation of SIRT3 and SUCLG2 protein in Adv-Cre-treated NCFs (Fig. S9C, D).

**Fig. 7:**
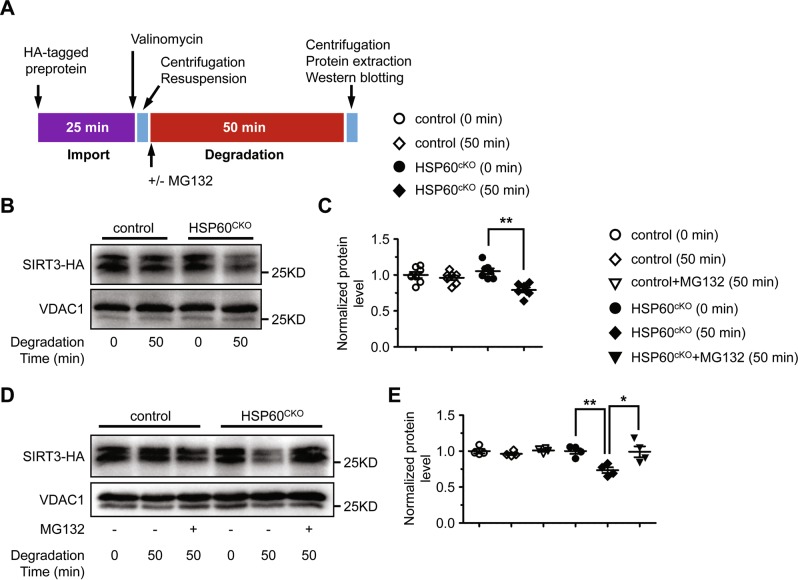
Measurement of protein import and degradation in isolated control and HSP60^CKO^ mitochondria. Mitochondria were isolated from control and HSP60^CKO^ hearts 6 weeks post tamoxifen injection. **a** Schematic diagram showing the procedure of mitochondrial protein import and degradation assay. Mitochondria were first incubated with HA-tagged pre-protein for 25 min. Import of HA-tagged mouse mitochondrial pre-protein and subsequent degradation was then assessed by western blotting of mitochondrial protein lysates extracted at 0 min and 50 min after the addition of valinomycin, respectively. **b**, **c** Western blotting (**b**) and quantitative analysis (**c**) showing that protein import was comparable between control and HSP60^CKO^ mitochondria while protein was degraded more rapidly in HSP60^CKO^ mitochondria. *n* = 6 per group. Data represent mean ± SEM. Significance was determined using the two-way ANOVA analysis with Bonferroni post-hoc test. **p < 0.01 versus HSP60^CKO^ (0 min). **d**, **e** Western blotting (**d**) and quantitative analysis (**e**) showing that MG132 could significantly impair protein degradation in HSP60^CKO^ mitochondria. *n* = 4 per group. Data represent mean ± SEM. Significance was determined using the two-way ANOVA analysis with Bonferroni post-hoc test. *p < 0.05, **p < 0.01 versus HSP60^CKO^ (50 min)

To further assess the contribution of cardiac mitochondria to protein degradation, we isolated mitochondria from control and HSP60^CKO^ hearts 6 weeks post tamoxifen injection, and performed protein import and degradation assays using the in vitro generated HA-tagged SIRT3 and HA-tagged NDUFA9 (Fig. 7a). NDUFA9 is a complex I component and was examined as an HSP60-independent protein since its expression was not significantly changed in HSP60CKO mitochondria as revealed by both proteomics and western blot analyses (Table S4; Fig. S10A, B). We found that import of HA-tagged SIRT3 or HA-tagged NDUFA9 into control and HSP60^CKO^ mitochondria was comparable (Fig. 7b, c; Fig. S9C), suggesting that mitochondrial protein import was not affected by deletion of HSP60. In other words, downregulation of HSP60-dependent proteins such as SIRT3 in mutant mitochondria was not a consequence of impaired mitochondrial protein import. Furthermore, no degradation of SIRT3 was observed in control mitochondria 50 min after import, whereas about 30% of imported protein had been degraded in mutant mitochondria within the same period (Fig. 7b, c). However, no degradation of NDUFA9 could be found in either control or HSP60^CKO^ mitochondria (Fig. S10C). Interestingly, addition of MG132 almost completely rescued SIRT3 degradation in mutant mitochondria (Fig. 7d, e). Taken together, these data strongly suggested that HSP60-dependent mitochondrial proteins could not be normally folded in mutant mitochondria and thus underwent protein degradation that mainly relied on LONP1.

## Discussion

In our present study, we used an inducible cardiac-specific αMHC-MerCreMer system to delete HSP60 in adult mouse cardiomyocytes. We found that deletion of HSP60 led to cardiac chamber dilation and left ventricular dysfunction, accompanied with a dramatic increase in the ratio of lung to body weight, and premature death, demonstrating that HSP60 deletion in adult cardiomyocytes resulted in dilated cardiomyopathy, which further led to heart failure and lethality (Fig. S11). Although knockout experiments have shown that HSP60 is essential for the survival of *Escherichia coli*, yeast, Drosophila, and mice [8, 15–20], the in vivo functions of HSP60 in cardiac physiology and diseases have not been well studied. Therefore, our present study provided evidence for the first time, to our knowledge, that HSP60 plays an essential role in maintaining normal cardiac morphology and function in mice. On the other hand, deletion of HSP60 in adult mouse cardiomyocytes was also associated with an increase of cell apoptosis and cardiac fibrosis in the hearts, which is also consistent with a previous study showing that reduced HSP60 expression could increase cell apoptosis in cultured neonatal cardiomyocytes [47].

The HSP60 chaperonin is located in the matrix and consists of both HSP60 and HSP10 subunits to form a barrel-shaped complex that has been suggested to facilitate folding of relatively small, soluble monomeric proteins [7, 8]. In *Escherichia coli*, ~250 different proteins were found to interact with GroEL (HSP60 from *Escherichia coli*), among which only about 85 proteins exhibited an obligate dependence on GroEL for folding, suggesting that the others can utilize either GroEL or the other chaperones for folding [48]. In mammals, the effects of HSP60 loss on mitochondrial protein homeostasis remain undefined. Deletion of HSP60 in mice resulted in a very early embryonic lethality [20], which makes it impossible to use homozygous mutant mice for further functional and biochemical studies. On the other hand, proteomic analysis of mitochondria from heterozygous mutant mice which express half level of HSP60 protein revealed that UQCRC1 and SOD2 were highly dependent on appropriate HSP60/HSP10 chaperone complex function [49, 50]. In our present study, we also performed proteomic analysis to identify effects of HSP60 deletion on mitochondrial protein homeostasis. We identified 2194 proteins in total, but only 676 proteins (30.8% of total identified proteins) were characterized as mitochondrial-localized proteins. This low percentage might largely arise from co-purifying contaminants that has been found to represent up to 75% of proteins detected in other MS/MS experiments [51]. Among these 676 identified mitochondrial-localized proteins, 121 proteins were downregulated and only eight proteins were upregulated in the HSP60^CKO^ hearts relative to their control counterparts. Western blot analysis of a selected subset of proteins confirmed proteomic results. Therefore, here we provided for the first time, at least to our knowledge, a list of mitochondrial proteins that require HSP60-mediated chaperone activity to maintain their protein levels in the mitochondria of adult mouse cardiomyocytes. We also believe it will be informative to determine which mitochondrial proteins bind directly to HSP60.

Most components of ETC complexes were identified in our proteomic analysis including 38 proteins of complex I, four proteins of complex II, nine proteins of complex III, 13 proteins of complex IV, and 16 proteins of complex V. In HSP60 mutant mitochondria, 10 ETC components were downregulated relative to controls, all belonging to complex I, demonstrating that complex I components are more susceptible to the loss of HSP60 than the other complex components. These selective effects on complex I components may reflect specificity for interaction with HSP60, or may reflect overall greater protein flux within complex I components. Complex I is the largest and first enzyme of the ETC, which is composed of 45 subunits and is essential for cellular energy production, providing about 40% of the proton motive force required for ATP synthesis [52].

Perturbed mitochondrial protein homeostasis in mutant mice first led to increased expression of cardiac fetal genes including *ANF* and *βMHC* 6 weeks post tamoxifen injection, suggesting that cardiac remodeling program was activated in mutant hearts even though UPR^mt^, mitochondrial dysfunction, and abnormalities in cardiac morphology and function were not observed in mutant hearts at this stage. Subsequently, UPR^mt^ was observed at 9 weeks post tamoxifen injection, indicating that mutant mitochondria were not able to degrade unfolded proteins, which started to accumulate, and was accompanied with impaired complex I and complex III activity, reduced mitochondrial membrane potential, increased mitochondrial ROS production, and the emergence of reduced cardiac systolic function in mutant hearts. Later on, expression of LONP1 and HTRA2 was increased in mutant hearts, likely as a compensatory mechanism but still could not prevent mutant hearts from developing chamber dilation and heart failure in the end.

Interestingly, downregulation of mitochondrial proteins in HSP60^CKO^ hearts 6 weeks post tamoxifen injection was not accompanied by reduced mRNA levels, suggesting that deletion of HSP60 does not affect transcription of these genes. Furthermore, mitochondrial import and degradation assays suggested that mitochondrial protein import was largely normal in mutant mitochondria, which was also consistent with the fact that we did not observe apparent changes in expression of the Translocases of Inner Mitochondrial Membrane (TIMMs) or the Translocases of Outer Mitochondrial Membrane (TOMMs) between control and mutant mitochondria in our proteomic data. We also did not observe any increases in expression of LONP1, CLPP, and HTRA2 in mutant mitochondria at the same stage. Our results further suggested that LONP1 may be the major protease responsible for protein degradation in mitochondria after HSP60 deletion, since addition of MG132, a LONP1 protease inhibitor [45, 46], rescued protein degradation in cell preparations as well as in mitochondrial preparations.

Taken together, our study demonstrated that HSP60 is required for maintaining normal cardiac morphology and function. Loss of HSP60 in adult cardiomyocytes altered mitochondrial protein homeostasis, impaired mitochondrial function, and eventually resulted in dilated cardiomyopathy and heart failure.

## Supplementary information

supplemental materials

## References

[1] Quiros PM, Mottis A, Auwerx J (2016). Mitonuclear communication in homeostasis and stress. Nat Rev Mol Cell Biol.

[2] Fosslien E (2003). Review: Mitochondrial medicine—cardiomyopathy caused by defective oxidative phosphorylation. Ann Clin Lab Sci.

[3] Palaniyandi SS, Qi X, Yogalingam G, Ferreira JC, Mochly-Rosen D (2010). Regulation of mitochondrial processes: a target for heart failure. Drug Disco Today Dis Mech.

[4] Mercer TR, Neph S, Dinger ME, Crawford J, Smith MA, Shearwood AM (2011). The human mitochondrial transcriptome. Cell.

[5] Pagliarini DJ, Calvo SE, Chang B, Sheth SA, Vafai SB, Ong SE (2008). A mitochondrial protein compendium elucidates complex I disease biology. Cell.

[6] Chacinska A, Koehler CM, Milenkovic D, Lithgow T, Pfanner N (2009). Importing mitochondrial proteins: machineries and mechanisms. Cell.

[7] Chakraborty K, Chatila M, Sinha J, Shi Q, Poschner BC, Sikor M (2010). Chaperonin-catalyzed rescue of kinetically trapped states in protein folding. Cell.

[8] Cheng MY, Hartl FU, Martin J, Pollock RA, Kalousek F, Neupert W (1989). Mitochondrial heat-shock protein hsp60 is essential for assembly of proteins imported into yeast mitochondria. Nature.

[9] Altieri DC, Stein GS, Lian JB, Languino LR (2011). TRAP-1, the mitochondrial Hsp90. Biochim Biophys Acta.

[10] Kang PJ, Ostermann J, Shilling J, Neupert W, Craig EA, Pfanner N (1990). Requirement for hsp70 in the mitochondrial matrix for translocation and folding of precursor proteins. Nature.

[11] Liu Q, Krzewska J, Liberek K, Craig EA (2001). Mitochondrial Hsp70 Ssc1: role in protein folding. J Biol Chem.

[12] Taipale M, Jarosz DF, Lindquist S (2010). HSP90 at the hub of protein homeostasis: emerging mechanistic insights. Nat Rev Mol Cell Biol.

[13] Nisemblat S, Yaniv O, Parnas A, Frolow F, Azem A (2015). Crystal structure of the human mitochondrial chaperonin symmetrical football complex. Proc Natl Acad Sci USA.

[14] Xu Z, Horwich AL, Sigler PB (1997). The crystal structure of the asymmetric GroEL-GroES-(ADP)7 chaperonin complex. Nature.

[15] Fayet O, Ziegelhoffer T, Georgopoulos C (1989). The groES and groEL heat shock gene products of Escherichia coli are essential for bacterial growth at all temperatures. J Bacteriol.

[16] Hohfeld J, Hartl FU (1994). Role of the chaperonin cofactor Hsp10 in protein folding and sorting in yeast mitochondria. J Cell Biol.

[17] Horwich AL, Low KB, Fenton WA, Hirshfield IN, Furtak K (1993). Folding in vivo of bacterial cytoplasmic proteins: role of GroEL. Cell.

[18] Reading DS, Hallberg RL, Myers AM (1989). Characterization of the yeast Hsp60 gene coding for a mitochondrial assembly factor. Nature.

[19] Perezgasga L, Segovia L, Zurita M (1999). Molecular characterization of the 5' control region and of two lethal alleles affecting the hsp60 gene in Drosophila melanogaster. FEBS Lett.

[20] Christensen JH, Nielsen MN, Hansen J, Fuchtbauer A, Fuchtbauer EM, West M (2010). Inactivation of the hereditary spastic paraplegia-associated Hspd1 gene encoding the Hsp60 chaperone results in early embryonic lethality in mice. Cell Stress Chaperon-.

[21] Hansen JJ, Durr A, Cournu-Rebeix I, Georgopoulos C, Ang D, Nielsen MN (2002). Hereditary spastic paraplegia SPG13 is associated with a mutation in the gene encoding the mitochondrial chaperonin Hsp60. Am J Hum Genet.

[22] Magen D, Georgopoulos C, Bross P, Ang D, Segev Y, Goldsher D (2008). Mitochondrial hsp60 chaperonopathy causes an autosomal-recessive neurodegenerative disorder linked to brain hypomyelination and leukodystrophy. Am J Hum Genet.

[23] Lin L, Kim SC, Wang Y, Gupta S, Davis B, Simon SI (2007). HSP60 in heart failure: abnormal distribution and role in cardiac myocyte apoptosis. Am J Physiol Heart Circ Physiol.

[24] Cheng Y, Sun J, Chen H, Adam A, Tang S, Kemper N (2016). Expression and location of HSP60 and HSP10 in the heart tissue of heat-stressed rats. Exp Ther Med.

[25] Lau S, Patnaik N, Sayen MR, Mestril R (1997). Simultaneous overexpression of two stress proteins in rat cardiomyocytes and myogenic cells confers protection against ischemia-induced injury. Circulation.

[26] Hollander JM, Lin KM, Scott BT, Dillmann WH (2003). Overexpression of PHGPx and HSP60/10 protects against ischemia/reoxygenation injury. Free Radic Biol Med.

[27] Lin KM, Lin B, Lian IY, Mestril R, Scheffler IE, Dillmann WH (2001). Combined and individual mitochondrial HSP60 and HSP10 expression in cardiac myocytes protects mitochondrial function and prevents apoptotic cell deaths induced by simulated ischemia-reoxygenation. Circulation.

[28] Shan YX, Liu TJ, Su HF, Samsamshariat A, Mestril R, Wang PH (2003). Hsp10 and Hsp60 modulate Bcl-2 family and mitochondria apoptosis signaling induced by doxorubicin in cardiac muscle cells. J Mol Cell Cardiol.

[29] Rodriguez CI, Buchholz F, Galloway J, Sequerra R, Kasper J, Ayala R (2000). High-efficiency deleter mice show that FLPe is an alternative to Cre-loxP. Nat Genet.

[30] Sohal DS, Nghiem M, Crackower MA, Witt SA, Kimball TR, Tymitz KM (2001). Temporally regulated and tissue-specific gene manipulations in the adult and embryonic heart using a tamoxifen-inducible Cre protein. Circ Res.

[31] Fang X, Stroud MJ, Ouyang K, Fang L, Zhang J, Dalton ND (2016). Adipocyte-specific loss of PPARgamma attenuates cardiac hypertrophy. JCI Insight.

[32] Tang H, Wang H, Lin Q, Fan F, Zhang F, Peng X (2017). Loss of IP3 receptor-mediated Ca(2+) release in mouse B cells results in abnormal B cell development and function. J Immunol.

[33] Ouyang K, Leandro Gomez-Amaro R, Stachura DL, Tang H, Peng X, Fang X (2014). Loss of IP3R-dependent Ca2+ signalling in thymocytes leads to aberrant development and acute lymphoblastic leukemia. Nat Commun.

[34] Fang X, Bogomolovas J, Wu T, Zhang W, Liu C, Veevers J (2017). Loss-of-function mutations in co-chaperone BAG3 destabilize small HSPs and cause cardiomyopathy. J Clin Invest.

[35] Lin Q, Zhao G, Fang X, Peng X, Tang H, Wang H (2016). IP3 receptors regulate vascular smooth muscle contractility and hypertension. JCI Insight.

[36] Sheikh F, Ouyang K, Campbell SG, Lyon RC, Chuang J, Fitzsimons D (2012). Mouse and computational models link Mlc2v dephosphorylation to altered myosin kinetics in early cardiac disease. J Clin Invest.

[37] Cooley N, Ouyang K, McMullen JR, Kiriazis H, Sheikh F, Wu W (2013). No contribution of IP3-R(2) to disease phenotype in models of dilated cardiomyopathy or pressure overload hypertrophy. Circ Heart Fail.

[38] Chen Y, Guo H, Xu D, Xu X, Wang H, Hu X (2012). Left ventricular failure produces profound lung remodeling and pulmonary hypertension in mice: heart failure causes severe lung disease. Hypertension.

[39] Smith AC, Robinson AJ (2009). MitoMiner, an integrated database for the storage and analysis of mitochondrial proteomics data. Mol Cell Proteom.

[40] Pellegrino MW, Nargund AM, Haynes CM (2013). Signaling the mitochondrial unfolded protein response. Biochim Biophys Acta.

[41] Haynes CM, Petrova K, Benedetti C, Yang Y, Ron D (2007). ClpP mediates activation of a mitochondrial unfolded protein response in C. elegans. Dev Cell.

[42] Ngo JK, Davies KJ (2007). Importance of the lon protease in mitochondrial maintenance and the significance of declining lon in aging. Ann N Y Acad Sci.

[43] Suzuki Y, Imai Y, Nakayama H, Takahashi K, Takio K, Takahashi R (2001). A serine protease, HtrA2, is released from the mitochondria and interacts with XIAP, inducing cell death. Mol Cell.

[44] van Loo G, van Gurp M, Depuydt B, Srinivasula SM, Rodriguez I, Alnemri ES (2002). The serine protease Omi/HtrA2 is released from mitochondria during apoptosis. Omi interacts with caspase-inhibitor XIAP and induces enhanced caspase activity. Cell Death Differ.

[45] Granot Z, Kobiler O, Melamed-Book N, Eimerl S, Bahat A, Lu B (2007). Turnover of mitochondrial steroidogenic acute regulatory (StAR) protein by Lon protease: the unexpected effect of proteasome inhibitors. Mol Endocrinol.

[46] Granot Z, Geiss-Friedlander R, Melamed-Book N, Eimerl S, Timberg R, Weiss AM (2003). Proteolysis of normal and mutated steroidogenic acute regulatory proteins in the mitochondria: the fate of unwanted proteins. Mol Endocrinol.

[47] Kirchhoff SR, Gupta S, Knowlton AA (2002). Cytosolic heat shock protein 60, apoptosis, and myocardial injury. Circulation.

[48] Kerner MJ, Naylor DJ, Ishihama Y, Maier T, Chang HC, Stines AP (2005). Proteome-wide analysis of chaperonin-dependent protein folding in Escherichia coli. Cell.

[49] Magnoni R, Palmfeldt J, Christensen JH, Sand M, Maltecca F, Corydon TJ (2013). Late onset motoneuron disorder caused by mitochondrial Hsp60 chaperone deficiency in mice. Neurobiol Dis.

[50] Magnoni R, Palmfeldt J, Hansen J, Christensen JH, Corydon TJ, Bross P (2014). The Hsp60 folding machinery is crucial for manganese superoxide dismutase folding and function. Free Radic Res.

[51] Calvo SE, Mootha VK (2010). The mitochondrial proteome and human disease. Annu Rev Genom Hum Genet.

[52] Hirst J (2013). Mitochondrial complex I. Annu Rev Biochem.

