# Supplementary material for: Deletion of heat shock protein 60 in adult mouse cardiomyocytes perturbs mitochondrial protein homeostasis and causes heart failure
Source: Cell Death Differ. 2019 Jun 17;27(2):587–600. doi: 10.1038/s41418-019-0374-x (PMC7205885; doi:10.1038/s41418-019-0374-x)
Supplement: Supplementary file 1 — supplemental materials [file 41418_2019_374_MOESM1_ESM.pdf]

Supplemental Figures and Figure Legends

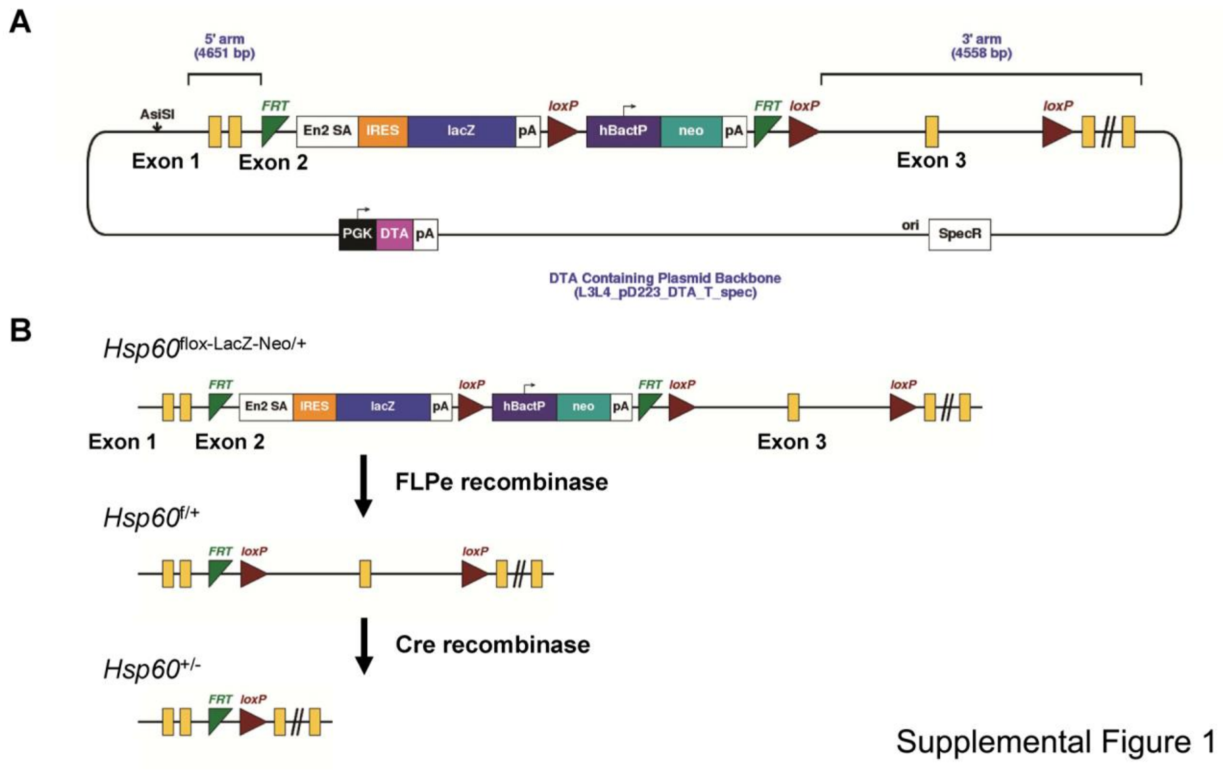

Supplemental Figure 1. Gene targeting strategy for mouse *Hsp60* gene. (**A**) Schematic diagram of targeting strategy for mouse *Hsp60* gene provided by the International Knockout Mouse Consortium. The targeting construct was generated by flanking exon 3 of *Hsp60* with loxP sites, while a LacZ-loxP-Neo cassette was flanked by FRT sites. (**B**) Schematic diagram of mouse crossing strategy to generate heterozygous *Hsp60* floxed and heterozygous *Hsp60* mutant alleles.

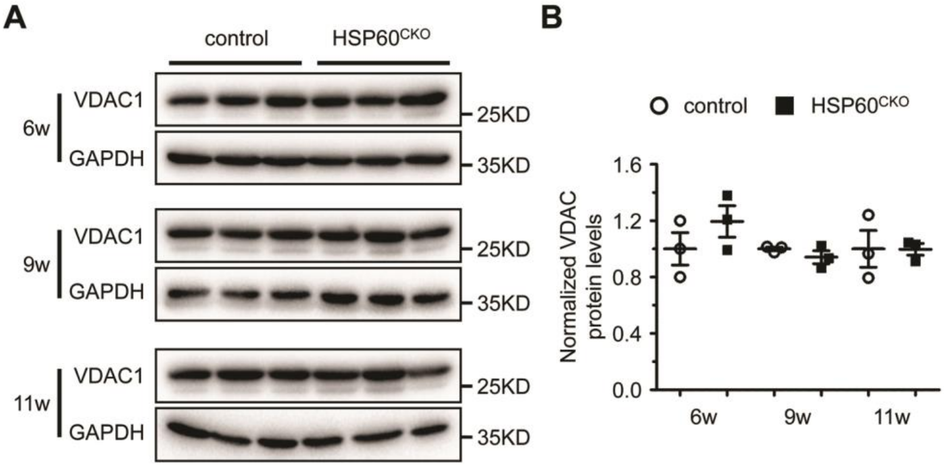

Supplemental Figure 2

Supplemental Figure 2. Expression of VDAC1 in control and HSP60^{CKO} hearts. (**A**) Western blot analysis of VDAC1 in control and HSP60^{CKO} hearts at 6 weeks (6w), 9 weeks (9w), and 11 weeks (11w) post tamoxifen injection. GAPDH was used as an internal loading control. (**B**) Quantitative analysis of VDAC1 protein levels. n = 3 mice per group. Data represent mean ± SEM. Significance was determined using the 2-way ANOVA analysis with Bonferroni post-hoc test. No significant difference in VDAC1 protein levels was observed between control and HSP60^{CKO} hearts at any stages indicated.

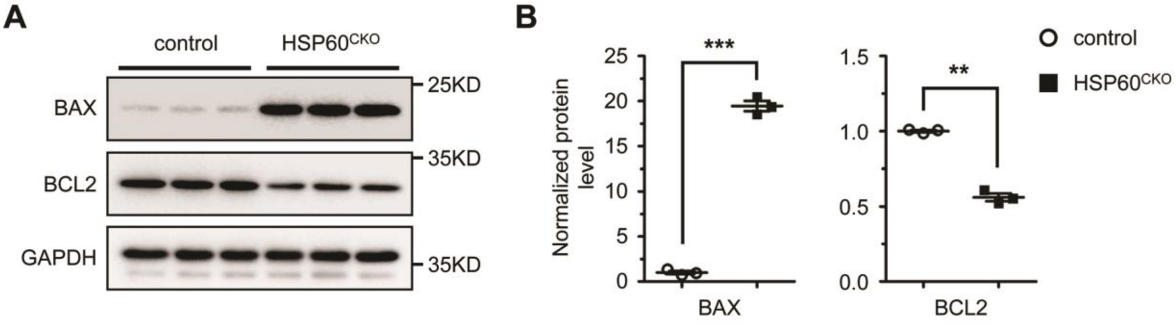

Supplemental Figure 3

Supplemental Figure 3. Expression of BAX and BCL2 in control and HSP60^{CKO} hearts. (**A**) Western blot analysis of BAX and BCL2 in control and HSP60^{CKO} hearts at 11 weeks post tamoxifen injection. GAPDH was used as an internal loading control. (**B**) Quantitative analysis of BAX and BCL2 protein levels. n = 3 mice per group. Data represent mean ± SEM. Significance was determined by the 2-tailed, unpaired Student's *t* test. $^{**}p < 0.01$, $^{***}p < 0.001$ versus control.

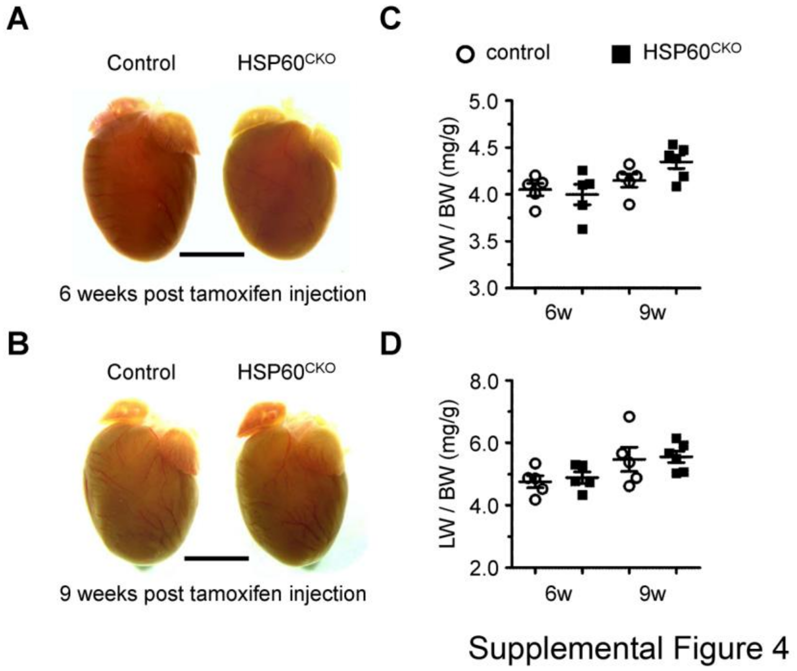

Supplemental Figure 4. Cardiac morphology and mass of HSP60^{CKO} mice 6 weeks and 9 weeks post tamoxifen injection. (**A**) and (**B**) Representative hearts of control and HSP60^{CKO} mice at 6 weeks (**A**) and 9 weeks (**B**) post tamoxifen injection. Scale bar, 3.0 mm. (**C**) and (**D**) Ratios of ventricle weight to body weight (VW / BW; **C**) and lung weight to body weight (LW / BW; **D**) of control and HSP60^{CKO} mice at 6 weeks (6w; n = 5 mice per group) and 9 weeks (9w; n = 5 mice for control group, n = 6 mice for HSP60^{CKO} group) post tamoxifen injection. Data represent mean ± SEM. Significance was determined by the 2-tailed, unpaired Student's *t* test.

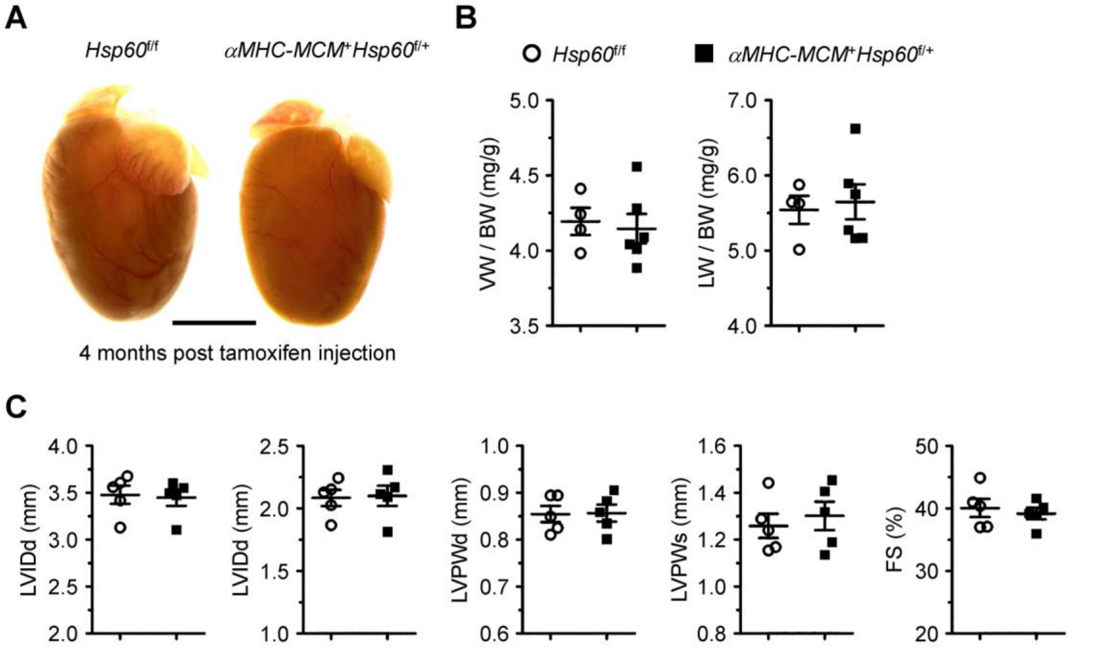

Supplemental Figure 5

Supplemental Figure 5. Morphological and functional analysis in *αMHC-MCM*$^{+}$*HSP60*$^{f/+}$ mice 4 months post tamoxifen injection. Male *Hsp60*$^{f/f}$ and *αMHC-MCM*$^{+}$*HSP60*$^{f/+}$ mice aged 7-8 weeks were treated with tamoxifen (20 mg/kg/day, 4 consecutive days) and were assessed with histological and echocardiographic analysis 4 months post tamoxifen injection. (**A**) Representative hearts of *Hsp60*$^{f/f}$ and *αMHC-MCM*$^{+}$*HSP60*$^{f/+}$ mice 4 months post tamoxifen injection. Scale bar, 3.0 mm. (**B**) Ratios of ventricle weight to body weight (VW/BW) and lung weight to body weight (LW / BW) of *Hsp60*$^{f/f}$ (n = 4) and *αMHC-MCM*$^{+}$*HSP60*$^{f/+}$ (n = 6) mice. Data represent mean ± SEM. Significance was determined by the 2-tailed, unpaired Student's *t* test. (**C**) Echocardiographic assessment of cardiac morphology and function. No significant difference in LVIDd, LVIDs, LVPWd, LVPWs, and FS was observed between *Hsp60*$^{f/f}$ and *αMHC-MCM*$^{+}$*HSP60*$^{f/+}$ mice 4 months post tamoxifen injection. n = 5 mice per group. Data represent mean ± SEM. Significance was determined by the 2-tailed, unpaired Student's *t* test.

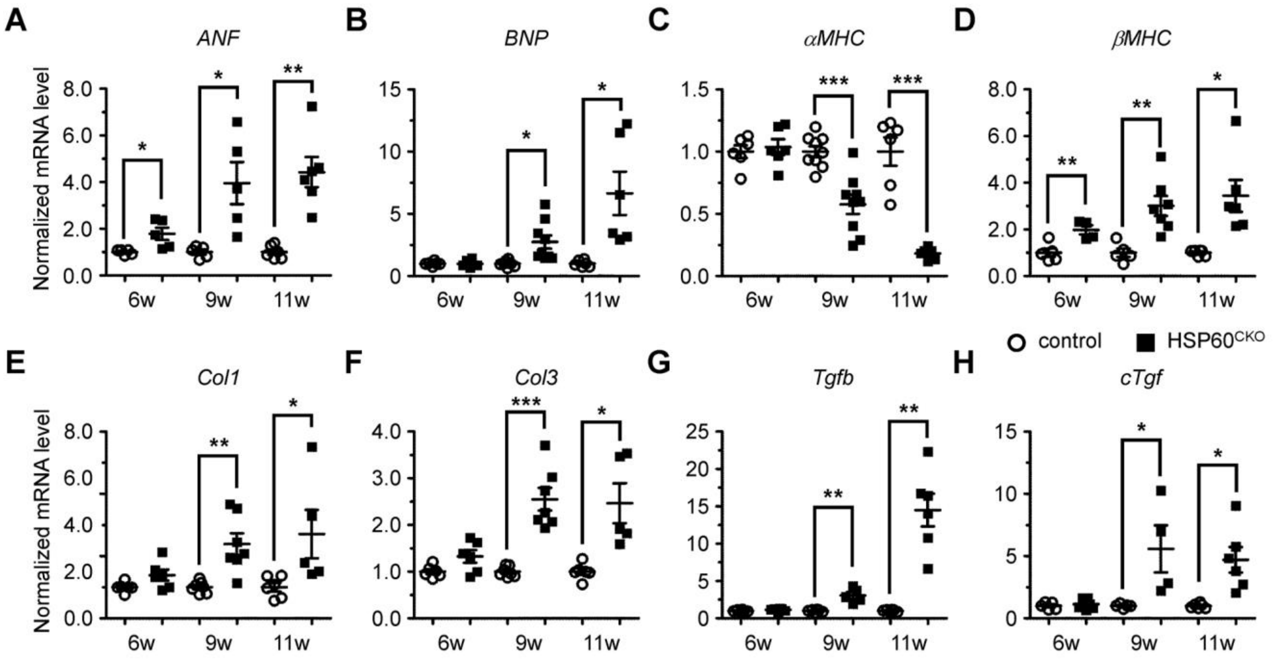

Supplemental Figure 6

Supplemental Figure 6. Expression of cardiac fetal and pro-fibrotic genes in control and HSP60^{CKO} mice. Quantitative RT-PCR analysis was performed to assess the expression of cardiac fetal genes including *ANF* (**A**), *BNP* (**B**), *αMHC* (**C**), and *βMHC* (**D**), and pro-fibrotic genes including *Col1* (**E**), *Col3* (**F**), *Tgfb* (**G**), and *cTgf* (**H**) in control and HSP60^{CKO} mice at 6 weeks (6w), 9 weeks (9w), and 11 weeks (11w) post tamoxifen injection. n = 5-7 mice per group. Data represent mean ± SEM. Significance was determined using the 2-way ANOVA analysis with Bonferroni post-hoc test. *p < 0.05, **p < 0.01, ***p < 0.001 versus control.

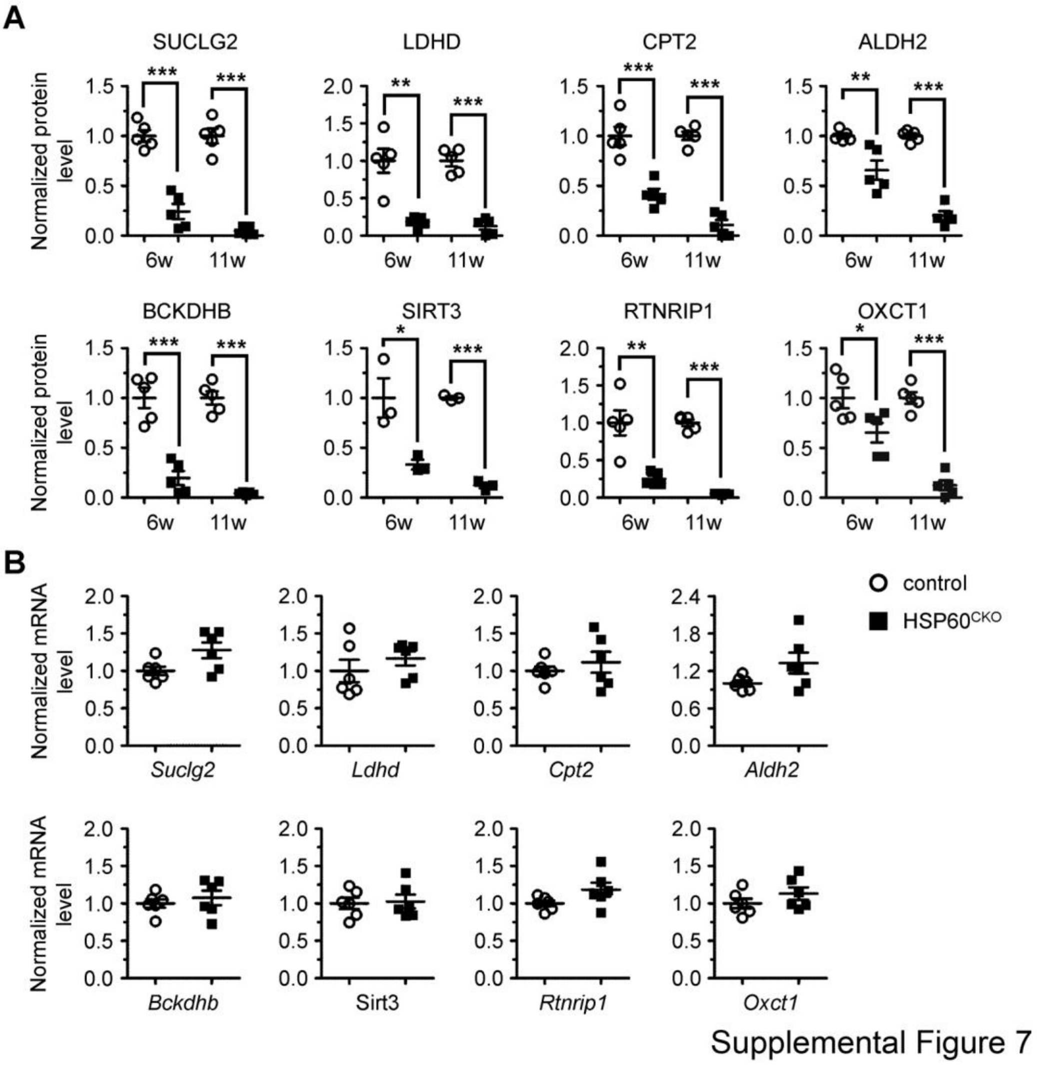

Supplemental Figure 7. Quantitative analysis of protein and mRNA levels of mitochondrial proteins in control and HSP60^{CKO} hearts. (**A**) Quantitative analysis of changes in protein levels of SUCLG2, LDHD, CPT2, ALDH2, BCKDHB, SIRT3, RTN4IP1, and OXCT1 between control and HSP60^{CKO} mitochondria at 6 weeks (6w) and 11 weeks (11w) post tamoxifen injection. n = 3-5 mice per group. Data represent mean ± SEM. Significance was determined using the 2-way ANOVA analysis with Bonferroni post-hoc test. *p < 0.05, **p < 0.01, ***p < 0.001 versus control. (**B**) Quantitative RT-PCR analysis of *Suclg2*, *Ldhd*, *Cpt2*, *Aldh2*, *Bckdhb*, *Sirt3*, *Rtn4ip1* and *Oxct1* mRNA levels in control and HSP60^{CKO} hearts 6 weeks post tamoxifen injection. n = 6 mice per group. Data represent mean ± SEM. Significance was determined by 2-tailed, unpaired Student's *t* test.

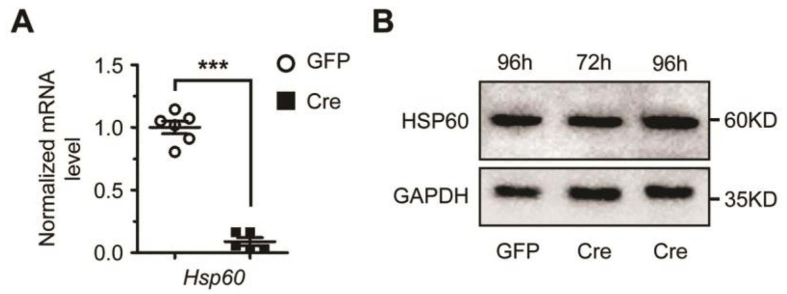

Supplemental Figure 8

Supplemental Figure 8. Measurement of HSP60 mRNA and protein levels in cultured neonatal mouse cardiomyocytes (NCMs). NCMs were isolated from neonatal HSP60$^{f/f}$ mouse hearts, cultured and treated with Adv-GFP and Adv-Cre, respectively. (**A**) Quantitative RT-PCR analysis showing that *Hsp60* mRNA level in cultured HSP60$^{f/f}$ NCMs treated with Adv-Cre (Cre; n = 5) was dramatically reduced compared with those treated with Adv-GFP (GFP; n = 6) at 72 hours (h) post the addition of adenovirus in the medium. Data represent mean ± SEM. Significance was determined by 2-tailed, unpaired Student's *t* test. ***p < 0.001 versus GFP. (**B**) Western blot analysis demonstrating that Adv-Cre treatment was not able to decrease HSP60 protein levels in cultured NCMs. Cell lysates were collected from cultured HSP60$^{f/f}$ NCMs treated with Adv-GFP for 96 hours, and with Adv-Cre for 72 and 96 hours, respectively. GAPDH was used as the protein loading control.

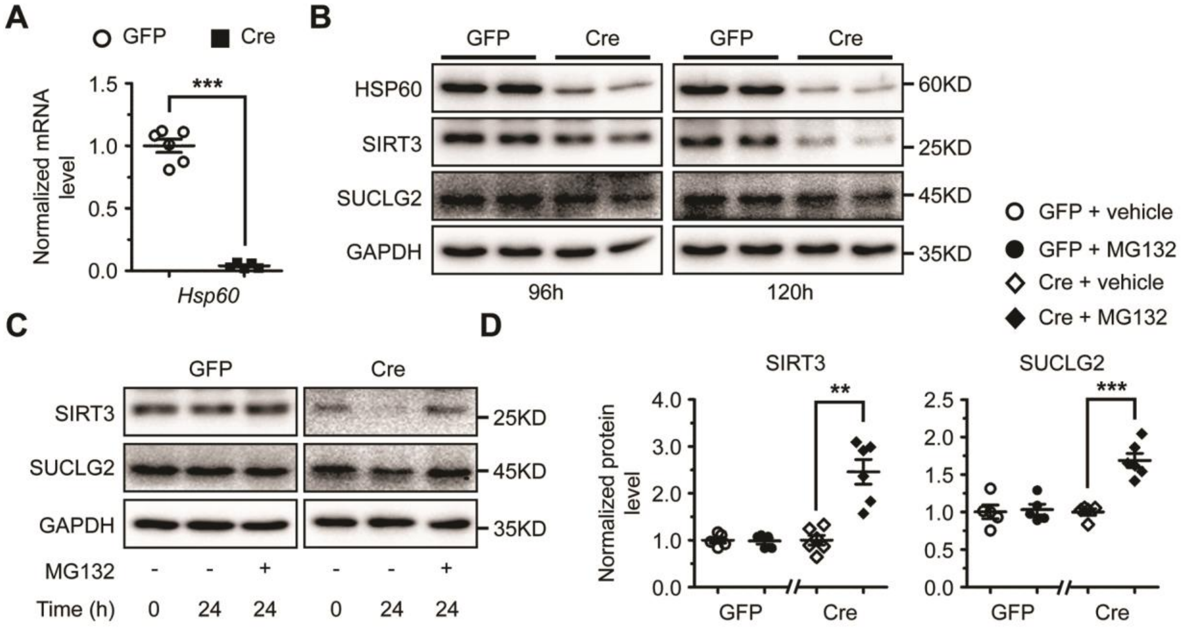

Supplemental Figure 9

Supplemental Figure 9. MG132 reduced downregulation of mitochondrial proteins by HSP60 deletion in cultured neonatal mouse cardiac fibroblasts (NCFs). NCFs were isolated from neonatal HSP60$^{f/f}$ mouse hearts, cultured and treated with Adv-GFP and Adv-Cre, respectively. (**A**) Quantitative RT-PCR analysis of *Hsp60* mRNA levels in cultured HSP60$^{f/f}$ NCFs that had been treated with Adv-Cre (Cre; n = 5) and Adv-GFP (GFP; n = 6) for 72 hours (h), respectively. Data represent mean ± SEM. Significance was determined by 2-tailed, unpaired Student's *t* test. ***p < 0.001 versus GFP. (**B**) Western blotting analysis of HSP60, SIRT3, and SUCLG2 in cultured HSP60$^{f/f}$ NCFs treated with Adv-GFP or Adv-Cre for 96 hours and 120 hours, respectively. GAPDH was used as the protein loading control. (**C**) The effect of MG132 on expression of SIRT3 and SUCLG2 in cultured HSP60$^{f/f}$ NCFs treated with Adv-GFP or Adv-Cre. Western blotting analysis (**C**) and quantitative analysis (**D**) revealed that addition of MG132 significantly increased protein levels of SIRT3 and SUCLG2 in cultured HSP60$^{f/f}$ NCFs treated with Adv-Cre. Data represent mean ± SEM. Significance was determined by 2-tailed, unpaired Student's *t* test. **p<0.01, ***p < 0.001 versus vehicle.

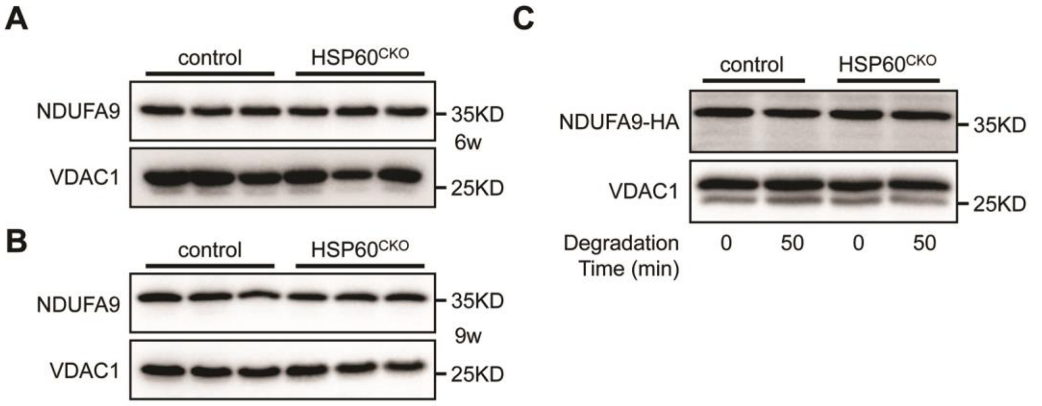

Supplemental Figure 10

Supplemental Figure 10. Measurement of NDUFA9 protein levels in control and HSP60CKO mitochondria. (**A**) and (**B**) Western blotting analysis of NDUFA9 in mitochondria isolated from control and HSP60^{CKO} hearts 6 weeks (6w, **A**) and 9 weeks (9w, **B**) post tamoxifen injection, respectively. VDAC1 was used as the protein loading control. (**C**) Mitochondria were isolated from control and HSP60^{CKO} hearts 6 weeks post tamoxifen injection. Import of HA-tagged mouse NDUFA9 pre-protein and subsequent degradation was then assessed by Western blotting analysis, showing comparable protein import and no degradation of NDUFA9 in HSP60^{CKO} mitochondria compared with control mitochondria.

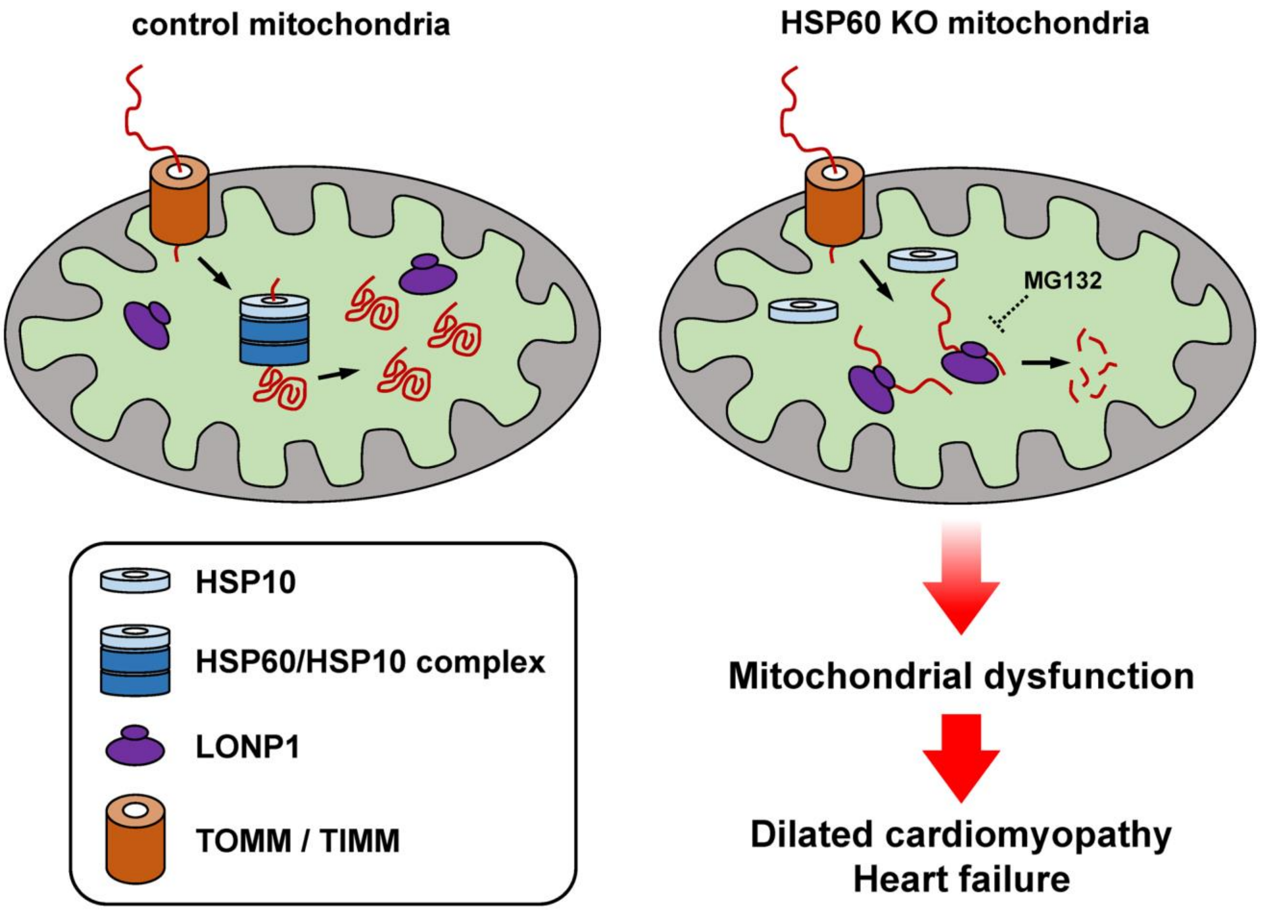

Supplemental Figure 11

Supplemental Figure 11. Schematic diagram showing the function of mitochondrial HSP60 in adult cardiac cells. In control mitochondria, nuclear-encoded mitochondrial precursor proteins are maintained in a relatively unfolded state, transported into mitochondria via the narrow pores formed by Translocases of Outer Mitochondrial Membrane (TOMMs) and Translocases of Inner Mitochondrial Membrane (TIMMs). Around 20% of these proteins are folded with assistance of the HSP60/HSP10 complex. In HSP60 knockout (KO) mitochondria, these proteins can be normally imported but are degraded too soon by the LON proteases such as LONP1, which can be blocked by MG132 treatment. Perturbed mitochondrial protein homeostasis in mutant hearts further leads to mitochondrial dysfunction and eventually results in dilated cardiomyopathy and heart failure.

Supplemental Table 1. Primers utilized for quantitative RT-PCR analyses.

Gene name	Forward primer (5' → 3')	Reverse primer (5' → 3')
Hsp60	AAAGATGGGGTCACTGTTGC	CATCACACCTCTCCGGATTT
mtHsp70	CTCTGGGAGGCGTCTTTACC	CGTTCCCCCTGACACACTTT
Chop	TCACTACTCTTGACCCTGCG	ACTGACCACTCTGTTTCCGT
Suclg2	GGAGTGAGAGTGCAGAGGTT	AACACCTCCTTTCAAACCGC
Ldhd	AACACGGGCATGATGAATCG	GCTCCGTGATCTGGTCCATA
Cpt2	CGCCCAGCTTCCATCTTTAC	CTGCCAGATACCGTAGAGCA
Aldh2	GCGTGGTCAATATCGTTCCC	GAAGAACAGGGCAAAGTGGG
Bckdhb	TTCCATGGCCCAAGAAAAGC	GGGGTGTCATATCCGCAAAC
Sirt3	TTTCATGTTGGCCAAGGAGC	GCTGTTACAAAGGTCCCGTG
Rtnrip1	CTCCTGAACATGGACCGACT	TTCCTGCATCCACCAGTTCT
Oxct1	ACAATGCAGGGGTTGACAAC	CCAGAGTCCCATACCCTGTG
Clpp	TGCAGTACATCCTGAACCCC	CTCTGCTTGGTGTGTTTGGC
ANP	TCGTCTTGGCCTTTTGGCT	TCCAGGTGGTCTAGCAGGTTCT
BNP	AAGCTGCTGGAGCTGATAAGA	GTTACAGCCCAAACGACTGAC
αMHC	TGAAAACGGAAAGACGGTGA	TCCTTGAGGTTGTACAGCACA
βMHC	CTACAGGCCTGGGCTTACCT	TCTCCTTCTAGACTTCCGC
Col1	TTCACCTACAGCACCCTTGT	GGTGGAGGGAGTTTACACGA
Col3	CAACCAGTGCAAGTGACCAA	GCACCATTGAGACATTTTGAAG
Tgfb	ACTGGAGTTGTACGGCAGTG	GGATCCACTTCCAACCCAGG
cTgf	GCATCTCCACCCGAGTTACC	AACTTGACAGGCTTGGCGAT
Gapdh	ATGACTCCACTCACGGCAAAT	TCCCATTCTCGGCCTTGAC

Supplemental Table 2. Antibodies utilized for Western blotting analysis.

Protein name	Vendor	Catalog no
HSP60	Santa Cruz Biotechnology	Sc-13966
GAPDH	Santa Cruz Biotechnology	Sc-32233
VDAC1	Sangon Biotech	D151112-0200
NDUFA9	Mito Science	MS111-SP/E0699
CLPP	Santa Cruz Biotechnology	Sc-47846
LONP1	Abcam	Ab103809
HTRA2	Santa Cruz Biotechnology	Sc-15467
SUCLG2	Santa Cruz Biotechnology	Sc-390818
LDHD	Santa Cruz Biotechnology	Sc-134673
CPT2	Pierce	PA5-12217
ALDH2	Santa Cruz Biotechnology	Sc-48837
BCKDHB	Santa Cruz Biotechnology	Sc-374630
SIRT3	Cell Signaling Technology	D22A3
RTN4IP1	Santa Cruz Biotechnology	Sc-514049
OXCT1	Santa Cruz Biotechnology	Sc-133988
BAX	Cell Signaling Technology	2772S
BCL2	Abgent	AP1303A

Supplemental Table 3. Mitochondrial ETC components identified by proteomic analysis in control and HSP60^{CKO} hearts.

Protein Accession	Protein description	Symbol	Complex	KO/Ct Ratio	P value	MW [Da]	pI	AASC [%]	MP	Score
O35683	NADH dehydrogenase [ubiquinone] 1 alpha subcomplex subunit 1	Ndufa1	I	0.731	0.169704193	9336	9.52	22.9	2	65
**P03888**	**NADH-ubiquinone oxidoreductase chain 1**	**mt-Nd1**	**I**	**0.587**	**0.00016967**	**37696**	**6.42**	**8.8**	**3**	**107**
P03893	NADH-ubiquinone oxidoreductase chain 2	mt-Nd2	I	0.838	0.402999597	41991	9.93	2	1	25
**P03899**	**NADH-ubiquinone oxidoreductase chain 3**	**mt-Nd3**	**I**	**0.479**	**0.988874922**	**14413**	**4.71**	**18.3**	**2**	**86**
P03911	NADH-ubiquinone oxidoreductase chain 4	mt-Nd4	I	0.917	0.260293171	55455	9.42	7.2	3	130
**P03921**	**NADH-ubiquinone oxidoreductase chain 5**	**mt-Nd5**	**I**	**0.669**	**0.144453636**	**74271**	**9.15**	**4.6**	**2**	**183**
P52503	NADH dehydrogenase [ubiquinone] iron-sulfur protein 6, mitochondrial	Ndufs6	I	0.837	5.61168E-05	15016	8.87	60.3	6	555
Q3UIU2	NADH dehydrogenase [ubiquinone] 1 beta subcomplex subunit 6	Ndufb6	I	0.747	4.16405E-05	17338	9.79	35.9	6	293
**Q8BK30**	**NADH dehydrogenase [ubiquinone] flavoprotein 3, mitochondrial**	**Ndufv3**	**I**	**0.653**	**3.46671E-05**	**13869**	**9.34**	**21.2**	**2**	**152**
Q8K3J1	NADH dehydrogenase [ubiquinone] iron-sulfur protein 8, mitochondrial	Ndufs8	I	0.684	0	27000	5.89	37.3	10	685
Q91VD9	NADH-ubiquinone oxidoreductase 75 kDa subunit, mitochondrial	Ndufs1	I	0.735	3.24901E-12	89918	5.51	62.4	38	3152
Q91WD5	NADH dehydrogenase [ubiquinone] iron-sulfur protein 2, mitochondrial	Ndufs2	I	0.751	2.50496E-09	58262	6.52	59.6	21	1125
Q91YT0	NADH dehydrogenase [ubiquinone] flavoprotein 1, mitochondrial	Ndufv1	I	0.684	3.24896E-12	57673	8.51	50.6	21	1289
Q99LC3	NADH dehydrogenase [ubiquinone] 1 alpha subcomplex subunit 10, mitochondrial	Ndufa10	I	0.683	3.24907E-12	47050	7.63	39.2	16	1130

Q99LY9	NADH dehydrogenase [ubiquinone] iron-sulfur protein 5	Pex19	I	0.769	2.30643E-11	16076	9.1	70.8	8	409
Q9CPP6	NADH dehydrogenase [ubiquinone] 1 alpha subcomplex subunit 5	Ndufa5	I	0.713	0.012538119	17075	7.82	59.5	8	375
Q9CQ54	NADH dehydrogenase [ubiquinone] 1 subunit C2	Ndufc2	I	0.711	1.50525E-06	16274	9.24	47.5	8	506
**Q9CQ75**	**NADH dehydrogenase [ubiquinone] 1 alpha subcomplex subunit 2**	**Ndufa2**	**I**	**0.61**	**7.39376E-06**	**12627**	**10.02**	**63.6**	**8**	**418**
Q9CQ91	NADH dehydrogenase [ubiquinone] 1 alpha subcomplex subunit 3	Ndufa3	I	0.817	0.001677443	10700	8.04	28.6	3	89
Q9CQC7	NADH dehydrogenase [ubiquinone] 1 beta subcomplex subunit 4	Ndufb4	I	0.826	0.008397679	17364	9.89	55	6	353
Q9CQH3	NADH dehydrogenase [ubiquinone] 1 beta subcomplex subunit 5, mitochondrial	Ndufb5	I	0.731	4.55413E-10	24503	9.43	21.2	4	294
Q9CQJ8	NADH dehydrogenase [ubiquinone] 1 beta subcomplex subunit 9	Ndufb9	I	0.774	4.23551E-05	25005	7.67	48	7	455
**Q9CQZ5**	**NADH dehydrogenase [ubiquinone] 1 alpha subcomplex subunit 6**	**Ndufa6**	**I**	**0.565**	**4.72108E-06**	**18023**	**10.1**	**46.6**	**9**	**411**
**Q9CQZ6**	**NADH dehydrogenase [ubiquinone] 1 beta subcomplex subunit 3**	**Ndufb3**	**I**	**0.647**	**1.96349E-09**	**13747**	**9.01**	**17.3**	**2**	**95**
**Q9CR21**	**Acyl carrier protein, mitochondrial**	**Ndufab1**	**I**	**0.636**	**0.000412077**	**19707**	**5.08**	**19.2**	**3**	**90**
Q9CR61	NADH dehydrogenase [ubiquinone] 1 beta subcomplex subunit 7	Ndufb7	I	0.718	2.59799E-06	19069	8.35	71.5	8	840
**Q9CXZ1**	**NADH dehydrogenase [ubiquinone] iron-sulfur protein 4, mitochondrial**	**Ndufs4**	**I**	**0.591**	**2.69329E-12**	**23725**	**10**	**40.6**	**7**	**538**
Q9D6J5	NADH dehydrogenase [ubiquinone] 1 beta subcomplex subunit 8, mitochondrial	Ndufb8	I	0.744	0.162030894	23523	6.15	29.6	4	500
Q9D6J6	NADH dehydrogenase [ubiquinone] flavoprotein 2, mitochondrial	Ndufv2	I	0.775	1.23712E-12	31047	7	42.3	9	990

Q9DC69	NADH dehydrogenase [ubiquinone] 1 alpha subcomplex subunit 9, mitochondrial	Ndufa9	I	0.706	8.10463E-15	47425	9.75	35	14	496
Q9DC70	NADH dehydrogenase [ubiquinone] iron-sulfur protein 7, mitochondrial	Ndufs7	I	0.765	1.98014E-05	27244	9.94	29.5	9	361
Q9DCJ5	NADH dehydrogenase [ubiquinone] 1 alpha subcomplex subunit 8	Ndufa8	I	0.671	3.24907E-12	24331	8.76	46.5	8	662
Q9DCS9	NADH dehydrogenase [ubiquinone] 1 beta subcomplex subunit 10	Ndufb10	I	0.827	0.000360115	24504	8.19	43.8	8	414
Q9DCT2	NADH dehydrogenase [ubiquinone] iron-sulfur protein 3, mitochondrial	Ndufs3	I	0.716	3.25417E-12	33281	6.67	38.8	11	929
**Q9Z1P6**	**NADH dehydrogenase [ubiquinone] 1 alpha subcomplex subunit 7**	**Ndufa7**	**I**	**0.651**	**9.99484E-12**	**15375**	**10.17**	**60.2**	**8**	**654**
Q8K2B3	Succinate dehydrogenase [ubiquinone] flavoprotein subunit, mitochondrial	Sdha	II	0.802	3.24907E-12	80956	7.06	44.9	27	2821
Q9CQA3	Succinate dehydrogenase [ubiquinone] iron-sulfur subunit, mitochondrial	Sdhb	II	0.824	2.10942E-15	38549	8.96	48.6	15	1068
Q9CXV1	Succinate dehydrogenase [ubiquinone] cytochrome b small subunit, mitochondrial	Sdhd	II	0.736	0.028917358	18434	9.3	4.4	1	36
Q9CZB0	Succinate dehydrogenase cytochrome b560 subunit, mitochondrial	Sdhc	II	0.951	0.552220466	20431	9.97	18.3	3	92
P00158	Cytochrome b	mt-Cytb	III	0.758	0.012976045	45873	7.79	4.2	3	48
P99028	Cytochrome b-c1 complex subunit 6, mitochondrial	Wbp11	III	0.712	3.97155E-08	12546	4.81	74.2	7	393
Q8R1I1	Cytochrome b-c1 complex subunit 9	Uqcr10	III	0.83	0.000231985	8816	9.16	26.6	1	537
Q9CQ69	Cytochrome b-c1 complex subunit 8	Uqcrq	III	0.838	0.000185529	10908	10.26	58.5	8	284
Q9CR68	Cytochrome b-c1 complex subunit Rieske, mitochondrial	Uqcrfs1	III	0.767	3.90588E-06	33530	8.91	48.5	13	1955
Q9CZ13	Cytochrome b-c1 complex subunit 1, mitochondrial	Uqcrc1	III	0.819	3.92479E-10	56654	5.81	33.1	14	3291

Q9D855	Cytochrome b-c1 complex subunit 7	Uqcrb	III	0.777	2.38179E-08	16498	9.1	64.9	10	638
Q9DB77	Cytochrome b-c1 complex subunit 2, mitochondrial	Uqcrc2	III	0.755	0	54679	9.26	45.7	18	3542
Q62425	Cytochrome c oxidase subunit NDUFA4	Ndufa4	IV	0.811	0.001532783	11383	9.52	62.2	7	312
P00405	Cytochrome c oxidase subunit 2	mt-Co2	IV	0.814	5.70888E-08	27505	4.6	35.2	7	855
P12787	Cytochrome c oxidase subunit 5A, mitochondrial	Cox5a	IV	0.777	3.24923E-12	18153	6.08	54.1	8	981
P17665	Cytochrome c oxidase subunit 7C, mitochondrial	Cox7c	IV	0.827	2.42036E-05	8474	11	36.5	3	600
P19536	Cytochrome c oxidase subunit 5B, mitochondrial	Cox5b	IV	0.827	5.98399E-08	15693	8.69	61.7	7	1346
P19783	Cytochrome c oxidase subunit 4 isoform 1, mitochondrial	Cox4i1	IV	0.817	3.24901E-12	23241	9.25	65.7	11	1746
P43023	Cytochrome c oxidase subunit 6A2, mitochondrial	Cox6a2	IV	0.765	5.26935E-06	11773	9.24	42.3	2	705
P43024	Cytochrome c oxidase subunit 6A1, mitochondrial	Cox6a1	IV	1.057	0.5825712	13261	9.97	14.4	1	23
P48771	Cytochrome c oxidase subunit 7A2, mitochondrial	Cox7a2	IV	0.932	0.438656918	10889	10.28	27.7	2	107
P56391	Cytochrome c oxidase subunit 6B1	Cox6b1	IV	0.764	1.11022E-16	12585	8.96	70.9	7	743
P56392	Cytochrome c oxidase subunit 7A1, mitochondrial	Cox7a1	IV	0.794	2.13653E-07	10470	9.83	28.8	2	471
P56393	Cytochrome c oxidase subunit 7B, mitochondrial	Cox7b	IV	0.872	0.021109888	10209	10.28	10	2	43
Q9CPQ1	Cytochrome c oxidase subunit 6C	Sf1	IV	0.698	6.30687E-06	10526	10.13	61.8	8	234
P00848	ATP synthase subunit a	mt-Atp6	V	1.169	0.586030407	26683	10.44	4	1	30
P03930	ATP synthase protein 8	mt-Atp8	V	0.988	0.458700438	9365	9.88	52.2	4	112
P56135	ATP synthase subunit f, mitochondrial	Atp5j2	V	0.89	0.000112155	12686	9.95	34.1	3	492
P56382	ATP synthase subunit epsilon, mitochondrial	Atp5e	V	0.801	0.021507931	7954	10.01	44.2	3	65
P56383	ATP synthase F(0) complex subunit C2, mitochondrial	Atp5g2	V	0.879	0.006853877	16726	10.36	4.8	1	141
P56480	ATP synthase subunit beta, mitochondrial	Atp5b	V	0.881	2.68687E-11	61765	5.19	68.6	27	12728
P97450	ATP synthase-coupling factor 6, mitochondrial	Atp5j	V	0.766	0.000181739	15468	9.36	46.3	5	494

Q03265	ATP synthase subunit alpha, mitochondrial	Atp5a1	V	0.868	0	67163	9.22	68.4	35	13692
Q06185	ATP synthase subunit e, mitochondrial	Hrk	V	0.822	1.32527E-08	10064	9.34	62	5	1155
Q91VR2	ATP synthase subunit gamma, mitochondrial	Atp5c1	V	0.892	2.78942E-08	39396	9.06	40.6	12	1378
Q9CPQ8	ATP synthase subunit g, mitochondrial	Atp5l	V	0.86	3.27027E-05	13938	9.74	60.2	7	436
Q9CQQ7	ATP synthase F(0) complex subunit B1, mitochondrial	Atp5f1	V	0.905	1.78436E-07	34544	9.11	37.1	13	1268
Q9CRA7	ATP synthase subunit s, mitochondrial	Atp5s	V	0.729	0.020368047	27669	8.33	14	3	68
Q9D3D9	ATP synthase subunit delta, mitochondrial	Atp5d	V	0.871	0.026334622	18735	5.03	41.1	4	2376
Q9DB20	ATP synthase subunit O, mitochondrial	Atp5o	V	0.77	3.26728E-12	28447	10	55.4	10	1338
Q9DCX2	ATP synthase subunit d, mitochondrial	Hoxc9	V	0.9	0.02991593	23149	5.52	71.4	13	1084

Supplemental Table 4. Downregulated and upregulated mitochondrial proteins in HSP60^{CKO} hearts.

Protein Accession	Protein description	Symbol	KO/Ct Ratio	P value	MW [Da]	pI	AASC [%]	MP	Score
Q9Z2I8	Succinyl-CoA ligase [GDP-forming] subunit beta, mitochondrial	Suclg2	0.228	3.2491E-12	55346	6.58	32.6	14	719
Q7TNG8	Probable D-lactate dehydrogenase, mitochondrial	Ldhd	0.268	0.00384888	55937	6.15	6.2	3	55
Q8R3F5	Malonyl-CoA-acyl carrier protein transacylase, mitochondrial	Mcat	0.275	0.009225728	46252	8.42	3.7	1	101
P63038	60 kDa heat shock protein, mitochondrial	Hspd1	0.278	0	73692	5.91	63.7	32	2204
Q99J25	rRNA methyltransferase 1, mitochondrial	Mrm1	0.29		37225	9.17	7.2	2	70
Q91WM2	Cat eye syndrome critical region protein 5 homolog	Hdhd5	0.294	0.042559176	49770	7.75	7.6	3	69
Q9CR24	Nucleoside diphosphate-linked moiety X motif 8, mitochondrial	Nudt8	0.315	0.02182006	24841	6.06	30	4	181
P52825	Carnitine O-palmitoyltransferase 2, mitochondrial	Cpt2	0.329	0	83670	8.59	24.6	16	1031
Q8R4N0	Citrate lyase subunit beta-like protein, mitochondrial	Clybl	0.361	4.56665E-12	44283	8.76	46.7	15	489
Q924D0	Reticulon-4-interacting protein 1, mitochondrial	Rtn4ip1	0.379	0.000230668	50331	9.29	23.5	8	240
Q6P3A8	2-oxoisovalerate dehydrogenase subunit beta, mitochondrial	Bckdhb	0.389	5.09247E-12	47776	6.27	21.5	7	299
P47738	Aldehyde dehydrogenase, mitochondrial	Aldh2	0.398	4.12842E-12	64119	7.53	34.1	16	504
Q9D7B6	Isobutyryl-CoA dehydrogenase, mitochondrial	Acad8	0.399	2.64632E-05	50830	8.46	26.4	10	332
Q9CXI0	2-methoxy-6-polyprenyl-1,4-benzoquinol methylase, mitochondrial	Coq5	0.4	0.002747672	41836	7.14	18	4	223
P85094	Isochorismatase domain-containing protein 2A, mitochondrial	Isoc2a	0.401	0.004712191	24578	8.25	44.7	6	161
Q8CH40	Nucleoside diphosphate-linked moiety X motif 6	Nudt6	0.406	0.003455679	38267	9.26	10.9	3	85
Q8BXN7	Protein phosphatase 1K, mitochondrial	Ppm1k	0.414	0.132321732	46620	5.96	8.6	2	51
Q9CWG8	NADH dehydrogenase [ubiquinone] complex I, assembly factor 7	Ndufaf7	0.421	0.004100258	54482	6.47	10.6	4	70

Q8R104	NAD-dependent protein deacetylase sirtuin-3	Sirt3	0.431	0.035495868	39454	7.05	11.4	4	142
Q9D6R2	Isocitrate dehydrogenase [NAD] subunit alpha, mitochondrial	Idh3a	0.436	0	46027	6.27	43.7	16	833
P70404	Isocitrate dehydrogenase [NAD] subunit gamma 1, mitochondrial	Idh3g	0.447	3.24879E-12	48199	9.17	26.2	11	360
Q9WU79	Proline dehydrogenase 1, mitochondrial	Prodh	0.448	0.000842218	74807	8.5	12.7	7	122
O88986	2-amino-3-ketobutyrate coenzyme A ligase, mitochondrial	Gcat	0.451	0.006889167	49368	6.92	8.2	4	83
Q9Z2Q5	39S ribosomal protein L40, mitochondrial	Mrpl40	0.455	0.119688442	28754	9.49	12.1	2	61
P50136	2-oxoisovalerate dehydrogenase subunit alpha, mitochondrial	Bckdha	0.457	5.30013E-10	54749	8.14	36	15	504
Q9JHI5	Isovaleryl-CoA dehydrogenase, mitochondrial	Ivd	0.462	0	52653	8.53	40.3	16	1127
Q8BMS4	Ubiquinone biosynthesis O-methyltransferase, mitochondrial	Coq3	0.468	1.33186E-08	45741	8.07	23.8	7	366
P16332	Methylmalonyl-CoA mutase, mitochondrial	Mut	0.468	2.22045E-16	94477	6.45	22.5	15	444
Q3TC72	Fumarylacetoacetate hydrolase domain-containing protein 2A	Usp17la	0.469	0.052281317	39650	8.42	8	2	84
P52196	Thiosulfate sulfurtransferase	Tst	0.47	0.000190003	37110	7.71	27.6	8	171
Q3UQ84	Threonine--tRNA ligase, mitochondrial	Tars2	0.477	1.09309E-07	88175	8.07	10.1	8	176
Q9R112	Sulfide:quinone oxidoreductase, mitochondrial	Sqor	0.477	4.86362E-05	58268	9.2	24.2	9	283
Q8VCW8	Acyl-CoA synthetase family member 2, mitochondrial	Acsf2	0.479	3.24446E-07	77070	8.44	15.1	10	216
Q99JY0	Trifunctional enzyme subunit beta, mitochondrial	Hadhb	0.479	3.2489E-12	59430	9.43	67.2	25	2789
P03899	NADH-ubiquinone oxidoreductase chain 3	mt-Nd3	0.479	0.988874922	14413	4.71	18.3	2	86
Q9JK42	[Pyruvate dehydrogenase (acetyl-transferring)] kinase isozyme 2, mitochondrial	Pdk2	0.486	0.000788931	51052	6.13	10.8	5	110
Q9CYW4	Haloacid dehalogenase-like hydrolase domain-containing protein 3	Hdhd3	0.487	0.087874456	30529	6.31	10.8	2	46
Q8BMS1	Trifunctional enzyme subunit alpha, mitochondrial	Hadha	0.488	1.11022E-16	98656	9.24	63.2	37	4218
P38060	Hydroxymethylglutaryl-CoA lyase, mitochondrial	Hmgcl	0.495	9.83246E-06	39714	8.7	26.8	7	349
Q9CR13	UPF0562 protein C7orf55 homolog	Fmc1	0.504		13947	10.1	13.3	1	131
Q8BWT1	3-ketoacyl-CoA thiolase, mitochondrial	Acaa2	0.504	3.2489E-12	47989	8.33	72.8	23	3579

B7ZMP1	Probable Xaa-Pro aminopeptidase 3	Xpnpep3	0.51	0.21546255	63685	7.6	3.8	2	67
Q9WTP7	GTP:AMP phosphotransferase AK3, mitochondrial	Ak3	0.513	4.00791E-14	29592	8.87	58.1	11	609
Q3TUH1	Phosphatidate cytidylyltransferase, mitochondrial	Tamm41	0.515	0.0387144	43016	9.07	6.8	2	82
P26443	Glutamate dehydrogenase 1, mitochondrial	Glud1	0.519	9.62174E-11	69661	8.05	36.9	19	925
P50171	Estradiol 17-beta-dehydrogenase 8	H2-Ke6	0.525	0.005952734	28862	6.1	39.8	7	353
O70571	[Pyruvate dehydrogenase (acetyl-transferring)] kinase isozyme 4, mitochondrial	Gramd1c	0.526	3.01468E-05	52179	6.61	18	7	175
O09174	Alpha-methylacyl-CoA racemase	Amacr	0.534	1.40515E-06	46603	7.01	23.4	8	327
Q91YY4	ATP synthase mitochondrial F1 complex assembly factor 2	Atpaf2	0.536	0.001168485	36647	5.94	7.6	2	67
Q8VE95	UPF0598 protein C8orf82 homolog	UPF0598	0.537	0.00789396	25573	9	24.8	4	101
Q6PE15	Mycophenolic acid acyl-glucuronide esterase, mitochondrial	Abhd10	0.54	0.302968485	38002	9.01	11.1	4	74
Q9DBL1	Short/branched chain specific acyl-CoA dehydrogenase, mitochondrial	Acadsb	0.541	7.61679E-07	55174	8	23.6	9	231
P55096	ATP-binding cassette sub-family D member 3	Abcd3	0.541	5.23099E-06	85793	9.32	13.5	10	230
P32020	Non-specific lipid-transfer protein	Scp2	0.551	1.61961E-06	70256	7.16	17.7	10	248
O88696	ATP-dependent Clp protease proteolytic subunit, mitochondrial	Clpp	0.552	0.000498087	32358	7.05	30.1	5	235
Q9D8P4	39S ribosomal protein L17, mitochondrial	Mrpl17	0.554	0.126501375	22585	9.83	9.7	2	49
P42925	Peroxisomal membrane protein 2	Pxmp2	0.554	0.008563875	24772	10.08	5.2	1	55
Q9CZS1	Aldehyde dehydrogenase X, mitochondrial	Aldh1b1	0.556	1.54412E-05	65420	6.59	16.6	8	136
P29758	Ornithine aminotransferase, mitochondrial	Oat	0.557	3.34269E-08	54910	6.19	16.9	7	293
Q07417	Short-chain specific acyl-CoA dehydrogenase, mitochondrial	Acads	0.558	3.24885E-12	50188	8.68	55.8	16	1567
Q9DCM0	Persulfide dioxygenase ETHE1, mitochondrial	Ethe1	0.564	0.023353445	30068	6.78	20.9	5	100
Q91ZE0	Trimethyllysine dioxygenase, mitochondrial	Tmlhe	0.565	0.329863199	55705	8.51	8.6	3	50
Q9CYK1	Tryptophan--tRNA ligase, mitochondrial	Wars2	0.565	0.074639046	47689	8.94	5.3	2	41
Q9CQZ5	NADH dehydrogenase [ubiquinone] 1 alpha subcomplex subunit 6	Ndufa6	0.565	4.72108E-06	18023	10.1	46.6	9	411
Q91YP0	L-2-hydroxyglutarate dehydrogenase, mitochondrial	L2hgdh	0.567	0.021800592	57738	8.59	17.5	9	170

Q60759	Glutaryl-CoA dehydrogenase, mitochondrial	Gcdh	0.578	6.43611E-07	53213	8.98	10.5	6	100
Q91V16	LYR motif-containing protein 5	Etfrf1	0.579	0.096090755	13364	9.73	18.6	2	30
Q8BWF0	Succinate-semialdehyde dehydrogenase, mitochondrial	Aldh5a1	0.584	0.000666277	64065	8.53	21	10	254
P03888	NADH-ubiquinone oxidoreductase chain 1	mt-Nd1	0.587	0.00016967	37696	6.42	8.8	3	107
P48410	ATP-binding cassette sub-family D member 1	Abcd1	0.59	0.002762308	88966	8.9	8.3	6	85
Q9CXZ1	NADH dehydrogenase [ubiquinone] iron-sulfur protein 4, mitochondrial	Ndufs4	0.591	2.69329E-12	23725	10	40.6	7	538
Q8R2Q4	Ribosome-releasing factor 2, mitochondrial	Gfm2	0.592	0.005989032	96019	6.01	7.7	5	187
Q80XL6	Acyl-CoA dehydrogenase family member 11	Acad11	0.596	9.58029E-07	98135	8.67	17.5	13	293
Q9D0D3	Poly(A) RNA polymerase, mitochondrial	Mtpap	0.596	0.048029861	74924	9.1	2.6	2	43
Q8C7H1	Methylmalonic aciduria type A homolog, mitochondrial	Mmaa	0.602	0.001976058	50943	9.45	18.3	7	124
Q99L13	3-hydroxyisobutyrate dehydrogenase, mitochondrial	Hibadh	0.603	6.07493E-05	40857	8.37	30.4	7	544
P45952	Medium-chain specific acyl-CoA dehydrogenase, mitochondrial	Acadm	0.605	3.24879E-12	54012	8.6	58.4	22	1660
Q3TLP5	Enoyl-CoA hydratase domain-containing protein 2, mitochondrial	Echdc2	0.607	0.044900077	34007	9.03	18.9	5	200
Q921S7	39S ribosomal protein L37, mitochondrial	Mrpl37	0.608	2.98098E-05	54210	9	25.8	9	232
Q9DAT5	Mitochondrial tRNA-specific 2-thiouridylase 1	Trmu	0.609	2.5581E-05	54426	8.67	10.8	3	152
Q9CQ75	NADH dehydrogenase [ubiquinone] 1 alpha subcomplex subunit 2	Ndufa2	0.61	7.39376E-06	12627	10.02	63.6	8	418
Q9D404	3-oxoacyl-[acyl-carrier-protein] synthase, mitochondrial	Oxsm	0.617	0.000175797	53807	6.6	15.3	6	169
Q91VT4	Carbonyl reductase family member 4	Cbr4	0.619	8.19887E-05	29465	9.71	37.7	7	391
Q9CQL5	39S ribosomal protein L18, mitochondrial	Mrpl18	0.619	0.005508025	23011	9.43	18.9	3	127
P53395	Lipoamide acyltransferase component of branched-chain alpha-keto acid dehydrogenase complex, mitochondrial	Dbt	0.619	8.72427E-10	62034	8.78	31.3	15	331
O35855	Branched-chain-amino-acid aminotransferase, mitochondrial	Bcat2	0.619	1.46882E-07	49481	8.61	23.2	8	342
Q9CQN7	39S ribosomal protein L41, mitochondrial	Mrpl41	0.62	0.003641617	18919	9.82	40	5	155
Q9DCA2	28S ribosomal protein S11, mitochondrial	Mrps11	0.621	2.12289E-05	22830	10.77	13.1	3	87

P09671	Superoxide dismutase [Mn], mitochondrial	Sod2	0.622	3.2489E-12	28711	8.8	33.3	8	1181
Q8JZS9	39S ribosomal protein L48, mitochondrial	Mrpl48	0.622	0.003843566	27890	9.45	13.7	3	55
Q9CQ06	39S ribosomal protein L24, mitochondrial	Mrpl24	0.624	0.095133363	28423	9.51	11.1	3	73
P56501	Mitochondrial uncoupling protein 3	Ucp3	0.625	0.009212928	37725	9.61	14	4	83
Q60936	Atypical kinase ADCK3, mitochondrial	Coq8a	0.628	3.3481E-12	80633	6.1	23.7	13	812
Q6NVE9	Protein phosphatase PTC7 homolog	Pptc7	0.629	0.117665057	35947	5.08	20.6	5	227
P08074	Carbonyl reductase [NADPH] 2	Cbr2	0.631	1.0647E-06	29722	9.1	21.7	4	170
O08756	3-hydroxyacyl-CoA dehydrogenase type-2	Hsd17b10	0.633	1.94825E-08	30724	8.53	43.7	9	740
O88844	Isocitrate dehydrogenase [NADP] cytoplasmic	Idh1	0.634	2.52691E-06	55981	6.73	15.9	6	157
Q8R0N6	Hydroxyacid-oxoacid transhydrogenase, mitochondrial	Adhfe1	0.636	1.34864E-08	56320	7.25	15.3	6	337
Q9CR21	Acyl carrier protein, mitochondrial	Ndufab1	0.636	0.000412077	19707	5.08	19.2	3	90
Q9CXT8	Mitochondrial-processing peptidase subunit beta	Pmpcb	0.638	0.000856349	59732	6.55	18.6	9	242
Q5SSK3	Transcription elongation factor, mitochondrial	Tefm	0.638	0.361703871	49121	9.56	8.5	3	71
Q9D0K2	Succinyl-CoA:3-ketoacid coenzyme A transferase 1, mitochondrial	Oxct1	0.639	3.24901E-12	65748	8.73	29.6	12	1635
Q9D051	Pyruvate dehydrogenase E1 component subunit beta, mitochondrial	Pdhb	0.642	3.24896E-12	43837	6.41	46	14	1598
Q99NB1	Acetyl-coenzyme A synthetase 2-like, mitochondrial	Acss1	0.644	3.07796E-09	81963	6.51	26.7	15	405
Q3UV70	[Pyruvate dehydrogenase [acetyl-transferring]]-phosphatase 1, mitochondrial	Pdp1	0.646	0.010602808	66983	6.2	3.5	2	54
Q9CQZ6	NADH dehydrogenase [ubiquinone] 1 beta subcomplex subunit 3	Ndufb3	0.647	1.96349E-09	13747	9.01	17.3	2	95
Q9CQ62	2,4-dienoyl-CoA reductase, mitochondrial	Decr1	0.649	5.86875E-10	42205	9.1	40.9	12	801
Q9Z1P6	NADH dehydrogenase [ubiquinone] 1 alpha subcomplex subunit 7	Ndufa7	0.651	9.99484E-12	15375	10.17	60.2	8	654
Q9WVL0	Maleylacetoacetate isomerase	Gstz1	0.651	0.016035016	27410	7.68	34.7	6	195
Q9D0Q7	39S ribosomal protein L45, mitochondrial	Mrpl45	0.652	0.645637897	40658	9.3	11.4	4	84
Q8BK30	NADH dehydrogenase [ubiquinone] flavoprotein 3,	Ndufv3	0.653	3.46671E-05	13869	9.34	21.2	2	152

	mitochondrial								
Q9DCM2	Glutathione S-transferase kappa 1	Gstk1	0.654	2.08935E-10	29697	8.97	48.2	10	404
P51174	Long-chain specific acyl-CoA dehydrogenase, mitochondrial	Acadl	0.654	3.24885E-12	54922	8.53	45.3	19	3049
P58064	28S ribosomal protein S6, mitochondrial	Mrps6	0.656	0.002742536	16419	9.51	29.6	4	72
Q9CQ40	39S ribosomal protein L49, mitochondrial	Mrpl49	0.656	1.50613E-05	21928	9.52	19.9	3	125
Q9QYR9	Acyl-coenzyme A thioesterase 2, mitochondrial	Acot2	0.659	6.293E-07	54437	6.88	43.5	15	666
Q8QZT1	Acetyl-CoA acetyltransferase, mitochondrial	Acat1	0.659	0	52463	8.71	45.5	17	2468
Q99J99	3-mercaptopyruvate sulfurtransferase	Mpst	0.66	0.012137747	36439	6.11	14.5	4	233
Q9JJL8	Serine--tRNA ligase, mitochondrial	Sars2	0.664	0.036774297	62460	8.03	7.5	3	95
P35486	Pyruvate dehydrogenase E1 component subunit alpha, somatic form, mitochondrial	Pdha1	0.667	0	49388	8.49	40.3	18	1475
P03921	NADH-ubiquinone oxidoreductase chain 5	mt-Nd5	0.669	0.144453636	74271	9.15	4.6	2	183
Q8VEA4	Mitochondrial intermembrane space import and assembly protein 40	Chchd4	1.523	0.05398594	17977	4.22	25.9	2	38
P28661	Septin-4	42982	1.533	0.060860661	62691	5.58	5	3	56
Q62167	ATP-dependent RNA helicase DDX3X	Ddx3x	1.556	0.000162664	81018	6.73	18	11	231
Q6ZWQ0	Nesprin-2	Syne2	1.65	0.194705577	904870	5.23	0.4	3	65
O55142	60S ribosomal protein L35a	Cfl2	1.662	0.000726222	15410	10.9	22.7	3	135
Q8BX02	KN motif and ankyrin repeat domain-containing protein 2	Kank2	1.667	0.059299434	97175	5.38	4.6	4	71
Q8BX10	Serine/threonine-protein phosphatase PGAM5, mitochondrial	Pgam5	1.831	0.028691612	34896	9.15	7.6	2	31
P28352	DNA-(apurinic or apyrimidinic site) lyase	Apex1	2.572	0.022208945	42971	8.04	11.4	3	52
